# Randomised, double-blind, placebo-controlled trials of non-individualised homeopathic treatment: systematic review and meta-analysis

**DOI:** 10.1186/s13643-017-0445-3

**Published:** 2017-03-24

**Authors:** Robert T. Mathie, Nitish Ramparsad, Lynn A. Legg, Jürgen Clausen, Sian Moss, Jonathan R. T. Davidson, Claudia-Martina Messow, Alex McConnachie

**Affiliations:** 1 Homeopathy Research Institute, London, UK; 2 Robertson Centre for Biostatistics, Institute of Health and Wellbeing, University of Glasgow, Glasgow, UK; 3 Department of Biomedical Engineering, University of Strathclyde, Glasgow, UK; 4 Karl und Veronica Carstens-Stiftung, Essen, Germany; 5 Department of Psychiatry and Behavioral Sciences, Duke University Medical Center, Durham, NC USA

**Keywords:** Non-individualised homeopathy, Meta-analysis, Randomised controlled trials, Sensitivity analysis, Systematic review

## Abstract

**Background:**

A rigorous systematic review and meta-analysis focused on randomised controlled trials (RCTs) of non-individualised homeopathic treatment has not previously been reported. We tested the null hypothesis that the main outcome of treatment using a non-individualised (standardised) homeopathic medicine is indistinguishable from that of placebo. An additional aim was to quantify any condition-specific effects of non-individualised homeopathic treatment.

**Methods:**

Literature search strategy, data extraction and statistical analysis all followed the methods described in a pre-published protocol. A trial comprised ‘reliable evidence’ if its risk of bias was low or it was unclear in one specified domain of assessment. ‘Effect size’ was reported as standardised mean difference (SMD), with arithmetic transformation for dichotomous data carried out as required; a negative SMD indicated an effect favouring homeopathy.

**Results:**

Forty-eight different clinical conditions were represented in 75 eligible RCTs. Forty-nine trials were classed as ‘high risk of bias’ and 23 as ‘uncertain risk of bias’; the remaining three, clinically heterogeneous, trials displayed sufficiently low risk of bias to be designated reliable evidence. Fifty-four trials had extractable data: pooled SMD was –0.33 (95% confidence interval (CI) –0.44, –0.21), which was attenuated to –0.16 (95% CI –0.31, –0.02) after adjustment for publication bias. The three trials with reliable evidence yielded a non-significant pooled SMD: –0.18 (95% CI –0.46, 0.09). There was no single clinical condition for which meta-analysis included reliable evidence.

**Conclusions:**

The quality of the body of evidence is low. A meta-analysis of all extractable data leads to rejection of our null hypothesis, but analysis of a small sub-group of reliable evidence does not support that rejection. Reliable evidence is lacking in condition-specific meta-analyses, precluding relevant conclusions. Better designed and more rigorous RCTs are needed in order to develop an evidence base that can decisively provide reliable effect estimates of non-individualised homeopathic treatment.

**Electronic supplementary material:**

The online version of this article (doi:10.1186/s13643-017-0445-3) contains supplementary material, which is available to authorized users.

## Background

Homeopathy is a system of medicine based fundamentally on the ‘Principle of Similars’: a substance capable of causing symptoms of illness in a healthy subject can be used as a medicine to treat similar patterns of symptoms experienced by an individual who is ill; homeopathic medicines are believed to stimulate a self-regulatory healing response in the patient [1]. There are several distinct forms of homeopathy, the main types being ‘individualised homeopathy’, ‘clinical homeopathy’ and ‘isopathy’. In *individualised homeopathy*, typically a single homeopathic medicine is selected on the basis of the ‘total symptom picture’ of a patient, including his/her mental, general and constitutional type. In *clinical homeopathy*, one or more homeopathic medicines are administered for standard clinical situations or conventional diagnoses; where more than one medicine is used in a fixed preparation, it is referred to as a ‘combination’ (devised by researchers) or ‘complex’ homeopathic medicine (available as an over-the-counter [OTC] proprietary formulation). *Isopathy* is the use of homeopathic dilutions from the causative agent of the disease itself, or from a product of the disease process, to treat the condition [1]: isopathic medicines include organisms and allergens prescribed on a basis that is different from individualised homeopathic prescribing in the classical sense.

To inform appropriate research development in homeopathy, the nature of its existing research evidence needs to be examined with rigour, objectivity and transparency. In a previous systematic review of randomised controlled trials (RCTs) of *individualised* treatment, we concluded there was a small, statistically significant, effect of the individually prescribed homeopathic medicines that was robust to sensitivity analysis based on reliable evidence; however, the low or uncertain quality of the evidence prevented a decisive conclusion [2].

In contrast to individualised treatment, placebo-controlled RCTs of *non*-individualised homeopathic treatment evaluate interventions that have involved the same, *standardised*, medication allocated to each and every participant randomised to homeopathy in a given trial: single homeopathic medicine, combination or complex homeopathic medicine, or isopathy. In this RCT context, none of these approaches involves matching a patient with the ‘total symptom picture’ of an individually prescribed homeopathic medicine: a pre-selected medicine is applied to the *typical* symptoms of a *clinical condition*. In the analysis reported in the present paper, we therefore regard all trials of non-individualised homeopathic treatment as, in effect, testing the same intervention. A study protocol for this systematic review has been published [3].

Three of five prior comprehensive reviews of homeopathy RCTs, reflecting the broad spectrum of clinical conditions that has been researched, reached the guarded conclusion that the homeopathic intervention probably differs from placebo [4–6]. The fourth such review concluded, ‘The results of our meta-analysis are not compatible with the hypothesis that the clinical effects of homeopathy are completely due to placebo’ [7], though the same authors later published supplementary analysis that weakened this conclusion [8]. The fifth of these global systematic reviews concluded there was “weak evidence for a specific effect of homoeopathic remedies…compatible with the notion that the clinical effects of homoeopathy are placebo effects” [9]. In their approach, however, each of these ‘global’ reviews has assessed collectively the findings for individualised *and* non-individualised homeopathy, a method we regard as inappropriate due to the distinction between the two types of intervention in the RCT context. There have been two systematic reviews, with meta-analysis, of *individualised* homeopathy trials: the first was published in 1998 [10], the most recent in 2014 [2]. A focused meta-analysis of non-individualised homeopathy RCTs has not previously been reported.

In order to synthesise the findings from placebo-controlled RCTs of non-individualised homeopathy we conducted an up-to-date systematic review and meta-analysis, testing the following null hypothesis: across the entire range of clinical conditions that have been researched, the main outcome of treatment using a non-individualised homeopathic medicine cannot be distinguished from that using placebo. An additional aim, further informing future research, was to quantify any effect of non-individualised homeopathic treatment for each clinical condition for which there is more than a single eligible RCT.

## Methods

Methods comply fully with the *PRISMA* 2009 Checklist (Additional file 1) and with our published protocol [3], which does not have a *PROSPERO* registration number.

### Search strategy, data sources and trial eligibility

We conducted a systematic literature search to identify RCTs that compared non-individualised homeopathy with a placebo, for any clinical condition [11]. Each of the following electronic databases was searched from its inception up to the end of 2011, with updated searches of the same databases up to the end of 2014: AMED; CAM-Quest®; CINAHL; Cochrane Central Register of Controlled Trials; Embase; Hom-Inform; LILACS; PubMed; Science Citation Index and Scopus. For the update, CORE-Hom® was also searched, using the term ‘randomised’ or ‘unknown’ in the *Sequence Generation* field.

The full electronic search strategy for PubMed (Cochrane Highly Sensitive Search Strategy) is given in our previous paper [11]: “((homeopath* *or* homoeopath*) *and* ((randomized controlled trial [pt]) *or* (controlled clinical trial [pt]) *or* (randomized [tiab]) *or* (placebo [tiab]) *or* (clinical trials as topic [mesh:noexp]) *or* (randomly [tiab]) *or* (trial [ti]))) *not* (animals [mh] *not* humans [mh])”.

As stated in our published protocol [3], we then excluded trials: of crossover design; of radionically prepared homeopathic medicines; of homeopathic prophylaxis; of homeopathy combined with other (complementary or conventional) intervention; for other specified reasons. The final explicit exclusion criterion was that there was obviously no blinding of participants and practitioners to the assigned intervention; for example, a trial described by the original authors as ‘single [i.e. patient-] blinded’ was automatically excluded. All remaining trials were eligible for systematic review.

### Outcome definitions

For each trial, and for the purposes of risk-of-bias assessment and meta-analysis, we identified a single ‘main outcome measure’ using a refinement of the approaches adopted by Linde et al. [7] and by Shang et al. [9]. Each trial’s ‘main outcome measure’ was identified based on the following hierarchical ranking order (consistent with the WHO International Classification of Functioning (ICF) linked to health condition [12]):

- Mortality
- Morbidity
  - ○ Treatment failure
  - ○ Pathology; symptoms of disease
- Health impairment (loss/abnormality of function, incl. presence of pain)
- Limitation of activity (disability, incl. days off work/school because of ill health)
- Restriction of participation (quality of life)
- Surrogate outcome (e.g. blood test data, bone mineral density).

We followed the WHO ICF system regardless of what measure may have been identified by the investigators as their ‘primary outcome’. In cases where, in the judgment of the reviewers, there were two or more outcome measures of equal greatest importance within the WHO ICF rank order, the designated ‘main outcome measure’ was selected randomly from those two or more options using the toss of coins or dice.

Unless otherwise indicated, the single end-point (measured from the start of the intervention) associated with the designated ‘main outcome measure’ was taken as the last follow-up at which data were reported for that outcome.

### Data extraction

Two reviewers (RTM and either JC, JRTD, LL, SM, NR or C-MM) identified the main outcome measure and then independently extracted data for each trial using a standard recording approach [3]. The data extracted per trial included, as appropriate: demographics of participants (gender, age range, medical condition); study setting; potency or potencies of homeopathic medicines; whether a pilot trial; ‘main outcome measure’ (see above) and measured end-point; funding source/s. The statistical items noted were whether statistical power calculation carried out; whether intention-to-treat (ITT) analysis; sample size and missing data for each intervention group. Discrepancies in the interpretation of data were discussed and resolved by consensus.

### Assessment of risk of bias

We used the domains of assessment as per the Cochrane risk-of-bias appraisal tool [13]. The extracted information enabled appraisal of freedom from risk of bias per domain: ‘Yes’ (low risk), ‘Unclear’ risk or ‘No’ (high risk). We applied this approach to each of the seven domains: sequence generation (domain I); allocation concealment used to implement the random sequence (II); blinding of participants and study personnel (IIIa); blinding of outcome assessors (IIIb); incomplete outcome data (IV); selective outcome reporting (V); other sources of bias (VI). The source of any research sponsorship (i.e. potential for vested interest) was taken into account for sub-group analysis (see below), but not in risk-of-bias assessment per se.

Reflecting appropriately the designated main outcome measure, we rated risk of bias for each trial across all seven domains and using the following classification [3]:

- Rating A = *Low risk of bias* in all seven domains.
- Rating B*x* = *Uncertain risk of bias* in *x* domains; low risk of bias in all other domains.
- Rating C*y.x* = *High risk of bias* in *y* domains; uncertain risk of bias in *x* domains; low risk of bias in all other domains.

#### Designating an RCT as ‘reliable evidence’

An ‘A’-rated trial was designated *reliable evidence*. We also designated a ‘B1’-rated trial *reliable evidence* if the uncertainty in its risk of bias was for one of domains IV, V or VI only (i.e. it was required to be judged free of bias for each of domains I, II, IIIA and IIIB) [3]; in tabulations and text below, this rating is shown as ‘B1* (*minimal risk of bias*)’.

### Study selection for meta-analysis

All RCTs that were included in the systematic review were potentially eligible for meta-analysis. If the original RCT paper did not provide adequate information on our selected main outcome measure to enable calculation of the SMD or the OR, we excluded the trial from the meta-analysis, and described the outcome as ‘not estimable’; consistent with Cochrane assessment criteria [13], such a trial was thus attributed *high risk of bias* in domain V.

### Statistical analysis

#### Data preparation

For a continuous main outcome measure, the mean, standard deviation (SD) and number of subjects were extracted for homeopathy and placebo groups and the unbiased standardised mean difference (SMD) calculated, so that a negative SMD reflected a difference in favour of homeopathy. We did not adjust values to compensate for any inter-group differences at baseline. For a dichotomous main outcome measure, the number of subjects with a favourable outcome and the total number of subjects in each group were extracted to enable calculation of the odds ratio (OR), with values greater than 1 reflecting a difference in favour of homeopathy.

For a given trial comprising more than two study groups, only the data concerning comparisons between non-individualised homeopathy and placebo were extracted from the paper. For a trial in which there were two or more homeopathy groups, those groups’ data were combined in analysis where relevant and feasible: for a dichotomous measure, combining data merely required summing the events and sample sizes; for a continuous measure, combining data was feasible only where SD was derivable^1^.

For the pooled meta-analysis, a single measure of effect size was required to enable pooling of all relevant trials: ORs were transformed to SMD using a recognised approximation method [14]. This is a deviation from the protocol, which stated that SMD would be transformed to OR, as in a previous paper [2]. SMD and OR are equally valid statistics. The reasoning behind using SMD instead of OR is that the latter is intuitively associated with a dichotomous outcome, whereas the former has a direct connection with ‘effect size’ and indicates that, for the meta-analysis, it has been derived via transformation from other measures (including OR). Whichever of these two metrics is used, their results are interchangeable and their interpretation is identical. ‘Effect size’ was interpreted as follows: SMD <0.40 = ‘small’; SMD 0.40 to 0.70 = ‘moderate’; SMD >0.70 = ‘large’ [14]. Via the SMD-to-OR transformation factor above [14], these values correspond, respectively, to: OR <2.10 = ‘small’; OR 2.10 to 3.60 = ‘moderate’; OR >3.60 = ‘large’, which we used for our previous paper [2].

#### Heterogeneity and publication bias

Due to the known clinical heterogeneity between studies, random-effects meta-analysis regression models [15] were used to derive pooled estimators and for sub-group / moderator analyses. Estimates were derived along with their 95% confidence intervals (CI) and *p* values. The *I* ^2^ statistic was used to assess the variability between studies: it gives the percentage of the total variability in the estimated effect size (which is composed of between-study heterogeneity plus sampling variability) that is attributable to heterogeneity. The *I* ^2^ statistic can take values between 0 and 100%: *I* ^2^ = 0% means that all of the heterogeneity is due to sampling error, and *I* ^2^ = 100% means that all variability is due to true heterogeneity between studies.

Funnel plots and Egger’s test of asymmetry [16, 17] and the ‘trim-and-fill’ method [18, 19] were used to assess the impact of publication bias.

All statistical analyses were carried out in *R* version 3.1.2 and using the *meta* package [20].

### Sensitivity analysis

The sensitivity analysis aimed to ascertain the impact of trials’ risk-of-bias rating on the pooled SMD: we examined the effect of cumulatively removing data from the meta-analysis by each trials’ rating, beginning with the lowest ranked ‘C’-rated trial/s.

### Sub-group analysis

Included in sub-group analysis was whether a trial: (a) had been included or not in previous meta-analysis [9]; (b) was a ‘pilot’ study; (c) necessitated our use of imputed data for the meta-analysis; (d) was free of vested interest; (e) investigated either an ‘acute’ or a ‘chronic’ clinical condition.

As was implicit in the study protocol [3], and as presented in a previous paper [2], we also included the following in sub-group analysis: (f) whether a trial had sample size that was greater or less than the median for those included in meta-analysis; (g) whether a trial used homeopathic medicine/s with potency ≥12C or <12C (12-times serial dilution of 1:100 starting solution), a concentration sometimes regarded as equivalent to the ‘Avogadro limit’ for molecular dose [21]; potency was defined as ‘mixed’ if a combination medicine in a given trial comprised a mixture of ≥12C and <12C potencies.

As recognised by Cochrane, some issues suitable for such analysis are identified during the review process itself [22]. Thus, we additionally carried out sub-group analysis depending on whether (h) a trial had investigated a combination, an OTC complex, an isopathic or a single remedy.

#### Disease-specific treatment effect of non-individualised homeopathy

Analysis was carried out by clinical condition, in cases where there were ≥2 RCTs with extractable main outcome. Analysis was additionally carried out by *category* of clinical condition, including each category for which there were data from ≥2 RCTs. RCT nomenclature for clinical conditions and their categories was previously characterised [11]^2^.

All sub-group analyses were conducted before and after removal of ‘C’-rated trials [2].

## Results

### Included studies

**Fig. 1:**
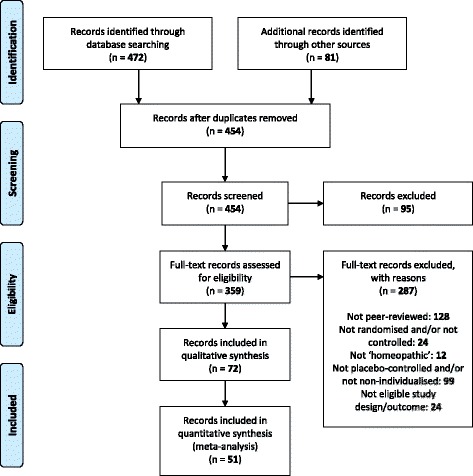
Updated *PRISMA* flowchart for all records published up to and including 2014

The *PRISMA* flowchart from the original comprehensive literature search (up to and including 2011) was published previously [11]. An updated *PRISMA* flowchart is given in Fig. 1, identifying a total of 553 records.^3^ Four-hundred and fifty-four remained after removal of duplicates. After excluding 95 due to type of record (book chapter, thesis, abstract and other minor article), Three-hundred and fifty-nine full-text records were then assessed for eligibility. Two-hundred and eighty-seven were excluded for the general reasons summarised in Fig. 1; 38 of these same 287 were excluded from the present systematic review for the additionally specified reasons shown in Additional file 2.^4^ The finally remaining 72 records (75 RCTs) were thus included in this systematic review; data were not extractable from 21 of those, leaving 51 records (54 RCTs) available for meta-analysis—see Additional file 2 for details of the 21 records excluded from meta-analysis.

### Characteristics of included studies

The 75 RCTs represent 48 different clinical conditions across 15 categories (Table 1). Each of 52 RCTs studied a condition that was acute in nature; each of 23 studied a chronic condition. Homeopathic potency was ≥12C in 29 trials, and not exclusively ≥12C for 7 trials (mix of >12C and <12C for 6 trials; unstated for 1 trial); potency was <12C in 39 trials. Seventeen trials were free of vested interest; 24 trials were *not* free of vested interest; 34 trials did not enable certainty in this assessment.

**Table 1:** Demographic data for 75 randomised controlled trials (RCTs) of non-individualised homeopathy: 21 excluded from meta-analysis shown by italics at first author's name

#	First author	Year	Category	Condition	Acute/ Chronic	Participants’ demographics	Study setting	Potency ≥12C	Hom. type	Funding source	Free of vested interest ^a^
A42	*Aabel*	2001	Allergy and Asthma	Seasonal allergic rhinitis	Acute	Patients treated for hay fever due to birch pollen allergy	Specialist outpatient department in Norway	Y	Isopathy	Research Council of Norway. Homeopathic remedy and placebo tablets were gift from DCG, Gothenburg, Sweden	N
A43	*Aabel*	2000	Allergy and Asthma	Seasonal allergic rhinitis	Acute	Patients treated for hay fever due to birch pollen allergy	Specialist outpatient department in Norway	Y	Isopathy	Research Council of Norway. Homeopathic remedy and placebo tablets were gift from DCG, Gothenburg, Sweden	N
A44	*Aabel*	2000	Allergy and Asthma	Seasonal allergic rhinitis	Acute	Patients treated for hay fever due to birch pollen allergy	Specialist outpatient department in Norway	Y	Isopathy	Research Council of Norway. Homeopathic remedy and placebo tablets were gift from DCG, Gothenburg, Sweden	N
A47	Baker	2003	Mental Disorder	Anxiety	Acute	Anxious students, aged 18-60 years	University, Australia	N	Single	‘Brauer Biotherapies Pty Ltd (Adelaide) manufactured the homeopathic preparation specifically for the study’	U
A48	Balzarini	2000	Dermatology	Radiodermatitis	Acute	Patients with dermatitis caused by radiotherapy for breast cancer	Rehabilitation and palliative care department, Italy	Mixed	Combn.	None stated	U
A49	Beer	1999	Obstetrics and Gynaecology	Induction of labour	Acute	Women (at least 18 years old) between 38 and 42 weeks’ gestation and cervical dilation (≤3 cm)	Akademisches Lehrkrankenhaus der RWTH Aachen	N	Single	None stated	U
A50	Belon	2006	Toxicology	Arsenic toxicity	Chronic	People at high risk of arsenic contamination	Rural village, India	Y	Single	Hom pharm	N
A51	*Belon*	2007	Toxicology	Arsenic toxicity	Chronic	People at high risk of arsenic contamination	Rural village, India	Y	Single	Hom pharm	N
A52	Bergmann (a)	2000	Obstetrics and Gynaecology	Female infertility: oligomenorrhoea	Chronic	Women, age 18 - 40	Universitäts-Frauenklinik Heidelberg	N	OTC complex	None stated	U
A52	Bergmann (b)	2000	Obstetrics and Gynaecology	Female infertility: amenorrhoea	Chronic	Women, age 18 - 40	Universitäts-Frauenklinik Heidelberg	N	OTC complex	None stated	U
A53	*Bernstein*	2006	Dermatology	Psoriasis	Acute	Patients with psoriasis	Dermatology Centre in the USA	N	Single	“Supported in part” by Apollo Pharmaceuticals Inc., manufacturers of Reliéva	N
A55	*Berrebi*	2001	Obstetrics and Gynaecology	Postpartum lactation	Acute	Women immediately after childbirth and who did not wish to breast-feed	Childbirth unit, university hospital, Toulouse, France	N	Combn.	Hom pharm	N
A56	Bignamini	1987	Cardiovascular	Hypertension	Chronic	Elderly males or females with hypertension	Two old people’s homes, Italy	Y	Single	“We thank Laboratoires Boiron for material provided”	U
A59	Cialdella	2001	Mental Disorder	Withdrawal of benzodiazepines	Chronic	Men and women aged >18 years, taking BZD for >3 mo.	GP practices in France (multi-centre)	N	OTC complex ^b^	External (hom pharm / govt.)	N
A60	Clark	2000	Musculoskeletal	Plantar fasciitis	Acute	Patients treated with heel pain due to fasciitis	Podiatry clinic, England	Y	Single	None stated	U
A272	Colau	2012	Obstetrics and Gynaecology	Menopausal syndrome	Chronic	Women aged ≥50 years; experienced amenorrhea for >12 months; spontaneously complained of hot flashes that had started <2 years previously	35 gynaecologist private practices in France	N	OTC complex	Hom pharm.	N
A61	Cornu	2010	Surgery and Anaesthesiology	Post-operative bleeding	Acute	Males or females, >18 years old, for whom elective aortic valve surgery planned	University hospital department, France	N	Combn.	Financially supported and assisted in design by Laboratoires Boiron, but ‘conducted in total independence’	U
A62	Diefenbach	1997	Respiratory Infection	Bronchitis	Acute	Patients (M/F) with bronchitis	4 doctors’ practices in Germany	N	OTC complex	None stated	U
A63	Ernst	1990	Cardiovascular	Varicose veins	Acute	Varicose veins in the legs	Rehabilitation clinic in Austria	N	OTC complex	None stated	U
A64	Ferley	1989	Respiratory Infection	Influenza	Acute	Age ≥12 years, flu-like symptoms, temp ≥38C	General practices in Rhône-Alpes region, France	Y	^a^Isopathy	None stated	U
A67	Frass	2005	Respiratory Infection	Tracheal secretions	Acute	Patients with accumulation of heavy secretions in the trachea	Intensive Care Unit at a university hospital in Austria	Y	Single	None stated	U
A68	Freitas	1995	Allergy and Asthma	Childhood asthma	Chronic	Children aged 12 mo to 12 years, with a mild to severe asthma crisis in the previous 6 mo.	Outpatient clinic in São Paulo, Brazil	N	Single	None stated	U
A69	*Friese*	2007	Ear, Nose and Throat	Sinusitis	Acute	Patients from 18 - 65 (m/f)	10 medical centres in Ukraine	N	OTC complex	None stated	U
A70	Friese	1997	Ear, Nose and Throat	Adenoid	Acute	Children (M/F) between age 4 and 10	Universitäts Kinderklinik Tübingen	Mixed	Combn.	Remedies were gift from hom pharm.	N
A74	Gerhard (a)	1998	Obstetrics and Gynaecology	Female infertility: amenorrhoea	Chronic	Women with secondary amenorrhoea	University hospital, Germany	N	OTC complex	Direct support from the company that provided the homeopathic medicine for the trial (senior author is company employee)	N
A74	Gerhard (b)	1998	Obstetrics and Gynaecology	Female infertility: luteal insufficiency	Chronic	Women with luteal insufficiency	University hospital, Germany	N	OTC complex	Direct support from the company that provided the homeopathic medicine for the trial (senior author is company employee)	N
A74	Gerhard (c)	1998	Obstetrics and Gynaecology	Female infertility: idiopathic	Chronic	Women with idiopathic infertility	University hospital, Germany	N	OTC complex	Direct support from the company that provided the homeopathic medicine for the trial (senior author is company employee)	N
A75	GRECHO	1989	Surgery and Anaesthesiology	Post-operative ileus	Acute	Men and women aged >18 years, having one of several stated operations	12 hospitals in France	Mixed	Combn.	Govt.	Y
A274	*Harrison*	2013	Mental Disorder	Insomnia	Chronic	Males aged 18-40, with chronic primary insomnia: at least 3 days per week for period of 1 mo to 10 years	University clinic	N	Combn.	University	Y
A76	*Hart*	1997	Surgery and Anaesthesiology	Post-operative pain	Acute	Patients with abdominal hysterectomy	Hospital, England	Y	Single	None stated	U
A78	*Hitzenberger*	2005	Cardiovascular	Hypertension	Chronic	Hypertensive patients (M/F)	Not explicitly mentioned	N	OTC complex	The study was funded, but it is not stated by whom	U
A79	Hofmeyr	1990	Obstetrics and Gynaecology	Postpartum pain	Acute	Postpartum women with episiotomy or perineal tearing requiring suture	University hospital, South Africa	N	Single ^b^	None stated	U
A80	*Jacobs*	2006	Gastroenterology	Childhood diarrhoea	Acute	Children with a history of acute diarrhoea	Two municipal clinics, Honduras	Y	Combn.	External (hom research foundation)	Y
A81	Jacobs	2007	Tropical Disease	Dengue fever symptoms	Acute	Patients over age 12 with a case definition of dengue	Two health centres, Honduras	Y	Combn.	External (hom research foundation); meds ‘furnished’ by hom pharm	U
A83	Kaziro	1984	Surgery and Anaesthesiology	Post-operative pain/swelling	Acute	Patients with extraction of impacted wisdom teeth	Dental hospital, England	Y	Single	None stated	U
A84	Khuda-Bukhsh	2005	Toxicology	Arsenic toxicity	Chronic	People at high risk of arsenic contamination	Rural village, India	Y	Single	Hom pharm.	N
A85	Khuda-Bukhsh	2011	Toxicology	Arsenic toxicity	Chronic	People at high risk of arsenic contamination	Rural village, India	Y	Single	Hom pharm.	N
A86	Kim	2005	Allergy and Asthma	Seasonal allergic rhinitis	Acute	Asthmatic people allergic to house dust mite	College of Naturopathic Medicine and Health Sciences, USA	N	Isopathy	Internal / External (hom pharm)	N
A88	*Kolia-Adam*	2008	Mental Disorder	Insomnia	Chronic	Males or females, 18-50 years, suffering from insomnia for > 1 year	University health clinic, South Africa	Y	Single	None stated	U
A89	Kotlus	2010	Surgery and Anaesthesiology	Post-operative bruising	Acute	Males undergoing upper blepharoplasty	University department of ophthalmology, USA	Y	Combn.	Supported in part by external foundation	Y
A91	*Labrecque*	1992	Dermatology	Warts	Chronic	Aged 6-59 years and at least 1 plantar wart untreated during previous 3 months	Hospital-based family medicine unit, Canada	Mixed	Combn.	Hospital pharmacy	Y
A92	Leaman	1989	Dermatology	Minor burns	Acute	Aged 15-60 years with minor burns within previous 6 h	Hospital Accident and Emergency Dept, England	Y	Single	None stated	U
A93	Lewith	2002	Allergy and Asthma	Allergic asthma	Chronic	Asthmatic people allergic to house dust mite	Hospital clinic, England	Y	Isopathy	Internal / External (charity) / Purchase from hom pharm	Y
A94	Lipman	1999	Miscellaneous	Snoring	Acute	People treated for snoring	ENT specialist clinic in the USA	N	OTC complex	None stated	U
A293	Malapane	2014	Ear, Nose and Throat	Tonsillitis	Acute	Children, aged 6-12 years, with acute viral tonsillitis	University department of homeopathy, South Africa	N	OTC complex	None stated	U
A95	*McCutcheon*	1996	Mental Disorder	Anxiety	Acute	Adults with above average anxiety	University, USA	N	OTC complex	Hom pharm	N
A275	Naidoo	2013	Allergy and Asthma	Allergic skin reaction	Acute	Males or females, aged 18 to 45; positive skin test; living with a cat for a period of ≥ 6 mo; suffering from allergy-like symptoms	University health training centre	N	Combn.	None stated	U
A100	Oberbaum	2001	Oral/Dental	Stomatitis	Acute	Cancer patients with stomatitis	Children’s Medical Centre in Israel	N	OTC complex	None stated	U
A101	Oberbaum	2005	Obstetrics and Gynaecology	Postpartum bleeding	Acute	Women after childbirth	Medical centre in Israel	Mixed	Combn.	Research foundation	Y
A103	Padilha	2011	Toxicology	Lead poisoning	Acute	Workers at risk of lead contamination	Clinic of battery plant, Brazil	Y	Single	Explicitly no funding source	Y
A104	Papp	1998	Respiratory Infection	Influenza	Acute	Age 12-60 years, specified flu-like symptoms, onset within last 24 h, temp ≥38C	General or specialist medical practices, Germany	Y	^a^ Isopathy	None stated	U
A105	Paris	2008	Surgery and Anaesthesiology	Post-operative analgesic intake	Acute	Male or female, aged 18-60 years, undergoing surgery of anterior cruciate ligamen	University hospital, France	N	Combn.	Hom pharm	N
A108	Rahlfs	1976	Gastroenterology	Irritable bowel syndrome	Acute	Patients, male or female, aged 20-60, with diagnosis of irritable bowel syndrome	12 general medical practices in Germany	N	Single ^b^	None stated	U
A109	Rahlfs	1978	Gastroenterology	Irritable bowel syndrome	Acute	Patients, male or female, aged 20-60, with diagnosis of irritable bowel syndrome	39 general medical practices in Germany	N	Single	External (hom research foundation)	Y
A277	*Razlog*	2012	Mental Disorder	ADHD	Chronic	School children, aged 5-11, diagnosed with ADHD	Primary schools in South Africa	N	Single	University	Y
A111	Reilly	1986	Allergy and Asthma	Seasonal allergic rhinitis	Acute	Patients aged over 5 with at least a 2-year history of seasonal rhinitis and current symptoms of grass pollen allergy	Two hospital clinics and 26 NHS general practitioners in the UK	Y	Isopathy	External: charity; govt.	Y
A112	Reilly	1994	Allergy and Asthma	Allergic asthma	Chronic	Patients aged over 16 with at least a 1-year history of asthma and reactive to inhaled allergens	Asthma outpatient clinic, Scotland	Y	Isopathy	External / purchase from hom pharm	Y
A113	Robertson	2007	Surgery and Anaesthesiology	Post-operative pain	Acute	Patients over the age of 18 undergoing tonsillectomy	Hospital, England	Y	Single	Internal / External (hom pharm)	N
A116	*Schmidt*	2002	Miscellaneous	Those benefited by reduced body weight	Acute	Fasting patients encountering static or increased body weight	Hospital for internal and complementary medicine, Germany	Y	Single	“The foundation of the Krankenhaus für Naturheilweisen funded the study, paying salaries or fees to contributors and collaborators.”	U
A117	*Seeley*	2006	Surgery and Anaesthesiology	Post-operative bruising	Acute	Female patients undergoing elective rhytidectomy	Hospital, Canada	Unknown	Single	Hom pharm.	N
A278	*Sencer*	2012	Oral/Dental	Mucositis	Acute	Patients aged 3-25 years, undergoing myeloablative haematopoietic SCT for malignant and non-malignant conditions	Oncology centres in USA, Canada and Israel	N	OTC complex	Government (NIH) grant	Y
A120	Singer	2010	Surgery and Anaesthesiology	Post-operative pain	Acute	Patients (M/F) age > = 18	2 centres	N	OTC complex	Financial support by producing company which ‘supplied the study medication’ but, by contractual agreement, had ‘no control over the flow of the study, the data analysis, or the decision when and where to publish the study findings’	Y
A122	*Stevinson*	2003	Surgery and Anaesthesiology	Post-operative pain/swelling	Acute	Aged 18–70 years undergoing elective hand surgery for carpal tunnel syndrome	Hospital, England	Y / N	Single ^b^	Hom. research foundation / drugs ‘supplied by’ hom pharm	U
A123	Taylor	2000	Allergy and Asthma	Perennial allergic rhinitis	Chronic	Patients with allergy to house dust mites, animals, pollens or foods	Specialist outpatient department in the UK	Y	Isopathy	External (hom and non-hom charities)	Y
A125	Tveiten	1991	Musculoskeletal	Muscle soreness	Acute	Male marathon runners, Norway	City of Oslo	Y	Single	None stated	U
A126	Tveiten	1998	Musculoskeletal	Muscle soreness	Acute	Marathon runners, Norway	City of Oslo	Y	Single	Research Council of Norway. Homeopathic remedy and placebo tablets were gift from hom pharm.	N
A128	Vickers	1998	Musculoskeletal	Muscle soreness	Acute	Long-distance runners, aged 18 or over, England	London community	Y	Single	Charity / drugs gifted by hom pharm	N
A130	*Weiser*	1994	Ear, Nose and Throat	Sinusitis	Chronic	Patients (M/F) with chronic sinusitis	11 ENT doctors’ practices in Germany	N	OTC complex ^b^	None stated	U
A131	Wiesenauer	1985	Allergy and Asthma	Seasonal allergic rhinitis	Acute	Patients treated for hay fever	Doctors’ practices in Germany	N	Single	External (hom pharm)	N
A132	*Wiesenauer*	1989	Ear, Nose and Throat	Sinusitis	Acute	Patients (M/F) with acute or chronic sinusitis	47 doctors’ practices	N	Combn. ^c^	Funded by two foundations	Y
A133	Wiesenauer	1990	Allergy and Asthma	Seasonal allergic rhinitis	Acute	M/F, all ages	54 doctors’ practices	N	Single	Funded by two foundations	Y
A134	Wiesenauer	1991	Rheumatology	Rheumatoid arthritis	Chronic	Patients (m/f), age 18 - 70, with chronic polyarthritis	6 doctors’ practices	N	OTC complex	None stated	U
A135	Wiesenauer	1995	Allergy and Asthma	Seasonal allergic rhinitis	Acute	Patients treated for hay fever	Doctors’ practices in Germany	N	Single	None stated	U
A136	Wolf	2003	Surgery and Anaesthesiology	Post-operative pain	Acute	Age 10 - 65 (M/F), varicose veins	Gefäßchirurgische Klinik in Berlin-Buch	N	Single	Remedies were gift from hom pharm.	N
A137	Zabolotnyi	2007	Ear, Nose and Throat	Sinusitis	Acute	Patients, aged 18-60 years, treated for acute maxillary sinusitis, with at least 8 d of symptoms	ENT specialist clinics, Ukraine	N	OTC complex	None stated	U

*Y* yes, *U* unclear, *N* no, *Combn.* combination, *M/F* male/female

^a^Vested interest: support (direct, through research sponsorship; indirect, via gifted medicines) from company that provided homeopathic medicines for the trial

^b^Single RCT comprising two homeopathy groups

^c^Single RCT comprising three homeopathy groups

### Summary of findings

For each trial, Table 2 includes details of the sample size, the identified main outcome measure (and whether dichotomous or continuous) and the study end-point. Seventeen trials were described in the original paper as a ‘pilot’ (or ‘preliminary’ or ‘feasibility’) study. A power calculation was carried out for 28 of the trials. ITT was the basis for analysis in 21 trials. Mean attrition rate was 14.6%. The main outcome variable was dichotomous in 25 studies and continuous in the other 50. The total sample size for the 54 meta-analysable trials was *5032*; the median sample size was 62.5 (inter-quartile range, 36 to 107). Meta-analysable studies included 45 different main outcome measures and for an end-point that ranged from 6 h to 6 months. Table 2 also indicates the 25 analysed trials in our study that we have in common with those included in the meta-analysis data reported by Shang et al. [9].

**Table 2:** Summary of findings table: 21 excluded from meta-analysis shown by italics at first author's name

#	First author	Year	Pilot	Power calc.	ITT sample	PP sample	PP sample > median (62.5)	Attrition rate %	Original ITT analysis	‘Main’ outcome identified	Nature of ‘main’ outcome	End-point
A42	*Aabel*	2001	N	N	51	51	N	0.0	N	Daily symptom score (VAS)	Continuous	10 days
A43	*Aabel*	2000	N	Y	70	66	Y	5.7	N	Daily symptom score	Continuous	32 days
A44	*Aabel*	2000	N	Y	80	73	Y	8.8	N	Daily symptom score (VAS)	Continuous	10 days
A47	Baker	2003	N	Y	?	44	N	?	N	Revised Test Anxiety (RTA) scale	Continuous	4 days
A48	^b^Balzarini	2000	N	N	66	61	N	7.6	N	Index of Total Severity during Recovery (re: skin colour, temp, oedema, pigmentation)	Continuous	7–8 weeks
A49	^b^Beer	1999	N	N	40	40	N	0.0	Y	Time between to regular uterine contractions	Continuous	7 h or induction of labour
A50	Belon	2006	N	N	43	43	N	0.0	N	Reversal in expression of antinuclear antibody titre	Dichotomous	1 month
A51	*Belon*	2007	Y	N	39	25	N	35.9	N	Blood arsenic concentration	Continuous	2 months
A52	^b^Bergmann (a)	2000	N	Y	?	37	N	?	N	Cycle normalisation	Dichotomous	3 months or 3 cycles
A52	^b^Bergmann (b)	2000	N	Y	?	30	N	?	Y	Cycle normalisation	Dichotomous	3 months or 3 cycles
A53	*Bernstein*	2006	N	N	200	171	Y	14.5	N	Psoriasis Area Severity Index	Continuous	12 weeks
A55	*Berrebi*	2001	N	N	71	71	Y	0.0	N	Mammary pain (VAS)	Continuous	4 days
A56	Bignamini	1987	N	N	34	32	N	5.9	N	(Systolic) Blood pressure	Continuous	4 weeks
A59	^b^Cialdella	2001	N	Y	96	61	N	36.5	Y	“Success rate” for clinical global impression	Dichotomous	30 days
A60	Clark	2000	Y	N	18	14	N	22.2	N	Daily pain (100 mm VAS)	Continuous	14 days
A272	Colau	2012	N	Y	108	101	Y	6.5	Y	Hot flash score	Continuous	12 weeks
A61	Cornu	2010	Y	Y	92	92	Y	0.0	Y	Cumulated blood loss at drain removal	Continuous	Up to 7 d
A62	^b^Diefenbach	1997	N	N	258	209	Y	19.0	Y	Treatment success (‘very good’ + ‘good’ results) – physician-assessed	Dichotomous	Up to 3 weeks
A63	^b^Ernst	1990	N	N	122 ^a^	122 ^a^	N	0.0	N	Venous filling time	Continuous	24 days
A64	^b^Ferley	1989	N	N	478	462	Y	3.3	N	Proportion of patients recovered (from 5 cardinal symptoms and from temp > 37.5)	Dichotomous	By 48 h
A67	Frass	2005	N	N	55	50	N	9.1	N	Total volume of tracheal secretions per day	Continuous	2 days
A68	^b^Freitas	1995	N	N	86	69	Y	19.8	N	Score of intensity, frequency and duration of symptoms	Continuous	6 months
A69	*Friese*	2007	N	N	144	68	Y	52.8	Y	Sinusitis symptoms score	Continuous	21 days
A70	^b^Friese	1997	N	Y	97	82	Y	15.5	N	Frequency of non-adenoidectomy (imputed)	Dichotomous	3 months
A74	Gerhard (a)	1998	N	Y	38	28	N	26.3	N	Frequency of pregnancy	Dichotomous	3 months
A74	Gerhard (b)	1998	N	Y	27	21	N	22.2	N	Frequency of pregnancy	Dichotomous	3 months
A74	Gerhard (c)	1998	N	Y	31	17	N	45.2	N	Frequency of pregnancy	Dichotomous	3 months
A75	GRECHO	1989	N	Y	300	300	Y	0.0	N	Number of hours from operation until first stool	Continuous	Up to c.100 h
A274	*Harrison*	2013	Y	N	34	28	N	17.6	N	Sleep onset latency	Continuous	28 days
A76	*Hart*	1997	N	N	93	73	Y	21.5	N	Frequency of improved pain score (VAS)	Dichotomous	Duration of 5 days
A78	*Hitzenberger*	2005	N	Y	?	?	-	?	N	Blood pressure	Continuous	6 weeks
A79	^b^Hofmeyr	1990	Y	N	162	161	Y	0.6	N	Daily questionnaire responses: those without moderate/severe perineal pain	Dichotomous	4 days
A80	*Jacobs*	2006	N	N	292	265	Y	9.2	Y	Duration of diarrhoea	Continuous	Up to 7 days
A81	Jacobs	2007	Y	N	60	58	N	1.7	N	No. of days until no pain or fever for at least two consecutive days	Continuous	Up to 1 weeks
A83	^b^Kaziro	1984	N	N	77	77	Y	0.0	N	Pain score (VAS): Numbers without moderate/severe pain (imputed)	Dichotomous	8 days
A84	Khuda-Bukhsh	2005	Y	N	55	55	N	0.0	N	Urine arsenic concentration (imputed)	Continuous	11 days
A85	Khuda-Bukhsh	2011	Y	N	28	14	N	50.0	N	Urine arsenic concentration	Continuous	2 months
A86	Kim	2005	Y	Y	40	34	N	15.0	Y	Rhinoconjunctivitis Quality-of-Life Questionnaire (RQLQ total symptoms)	Continuous	4 weeks
A88	*Kolia-Adam*	2008	N	N	30	30	N	0.0	N	Hours of sleep per night	Continuous	8 weeks
A89	Kotlus	2010	N	N	60	57	N	5.0	N	Area of ecchymosis	Continuous	7 days
A91	*Labrecque*	1992	N	Y	174	162	Y	6.9	N	Proportion of pts with healed warts (physician assessment)	Dichotomous	18 weeks
A92	^b^Leaman	1989	N	N	34	34	N	0.0	N	Pain (0-10 VAS) - area-under-the-curve	Continuous	6 h
A93	Lewith	2002	N	N	242	202	Y	16.5	Y	Asthma VAS (imputed)	Continuous	16 weeks
A94	Lipman	1999	N	N	101	90	Y	10.9	N	Average snoring score computed from responses to Snore Diary over last 5 nights of 10	Continuous	Duration of 10 days
A293	Malapane	2014	Y	N	30	30	N	0.0	N	Tonsillitis pain score (Wong-Baker FACES)	Continuous	6 days
A95	*McCutcheon*	1996	N	N	77	58	N	24.7	N	State Anxiety score	Continuous	Duration of 15 days
A275	Naidoo	2013	Y	N	30	30	N	0.0	N	Wheal diameter	Continuous	4 weeks
A100	Oberbaum	2001	N	N	32	30	N	6.3	Y	Area-under-the-curve score for stomatitis symptoms (severity and duration) (imputed)	Continuous	14 days minimum
A101	^b^Oberbaum	2005	Y	N	45	40	N	11.1	Y	Venous haemoglobin	Continuous	72 h postpartum
A103	Padilha	2011	N	N	131	120	Y	8.4	Y	Proportion of workers with Pb decrease of at least 25% (imputed)	Dichotomous	30 days
A104	^b^Papp	1998	N	N	372	334	Y	10.2	N	Proportion of patients with physician-assessed recovery in health (i.e. ‘no symptoms’)	Dichotomous	By 48 h
A105	Paris	2008	N	Y	131	105	Y	19.8	Y	Proportion patients with cumulated consumption of morphine < 10 mg/day (imputed)	Dichotomous	24 h post-op
A108	^b^Rahlfs	1976	Y	Y	?	63	Y	?	N	Improvement of irritable bowel syndrome (scale 1 + 2)	Dichotomous	14 days
A109	^b^Rahlfs	1978	Y	N	119	85	Y	28.6	N	Improvement of irritable bowel syndrome (scale 3 + 4)	Dichotomous	15 days
A277	*Razlog*	2012	Y	N	20	18	N	10.0	N	Conner’s PSQ (‘Impulsivity and/or hyperactivity’ category)	Continuous	3 weeks
A111	^b^Reilly	1986	N	Y	158	109	Y	31.6	N	Propn. with improvement in daily overall VAS score (imputed)	Dichotomous	5 weeks
A112	^b^Reilly	1994	N	Y	28	24	N	14.3	Y	Propn. with improvement in daily overall VAS score	Dichotomous	4 weeks
A113	Robertson	2007	N	Y	190	111	Y	41.6	N	Tonsillectomy pain (VAS) score	Continuous	14 days
A116	*Schmidt*	2002	N	Y	208	194	Y	6.7	Y	Reduction of body weight	Continuous	3 days
A117	*Seeley*	2006	N	N	29	26	N	10.3	N	Area of ecchymosis	Continuous	10 days
A278	*Sencer*	2012	N	Y	195	106	Y	45.6	N	Sum of Walsh scores for mucositis	Continuous	Up to 20 days post-transplant
A120	Singer	2010	N	Y	80	79	Y	1.3	Y	Area-under-the-curve pain score	Continuous	14 days
A122	*Stevinson*	2003	Y	N	64	62	N	3.1	Y	Pain (Short Form McGill Pain Questionnaire	Continuous	14 days
A123	^b^Taylor	2000	N	Y	51	50	N	2.0	Y	Daily overall VAS score (imputed)	Continuous	3–4 weeks
A125	Tveiten	1991	N	N	44	36	N	18.2	N	Muscle soreness (VAS) (imputed)	Continuous	3 days
A126	^b^Tveiten	1998	N	N	?	46	N	?	N	Muscle soreness (VAS)	Continuous	3 days
A128	^b^Vickers	1998	N	Y	?	400	Y	?	Y	Muscle soreness (VAS)	Continuous	2 days
A130	*Weiser*	1994	N	Y	173	155	Y	10.4	N	Sinusitis score	Continuous	5 months or on relapse
A131	^b^Wiesenauer	1985	N	N	106	74	Y	30.2	N	Symptom relief (nasal): ‘Symptom-free’ + ‘Obvious relief’	Dichotomous	4 weeks
A132	*Wiesenauer*	1989	N	N	221	152	Y	31.2	N	Sinusitis score	Continuous	3–4 weeks
A133	^b^Wiesenauer	1990	N	N	243	171	Y	29.6	N	Symptom relief (nasal): ‘Symptom-free’ + ‘Obvious relief’	Dichotomous	Approx 5 weeks
A134	Wiesenauer	1991	N	N	176	106	Y	39.8	N	Treatment success	Dichotomous	12 weeks
A135	^b^Wiesenauer	1995	N	N	132	120	Y	9.1	N	Symptom relief (nasal): ‘Symptom-free’ + ‘Obvious relief’	Dichotomous	4 weeks
A136	Wolf	2003	Y	N	60	59	N	1.7	N	Haematoma area	Continuous	2 weeks
A137	Zabolotnyi	2007	N	Y	113	113	Y	5.3	Y	Sinusitis severity score cf. Day 0 (imputed)	Continuous	7 days

*ITT* intention to treat, *PP* per protocol, *Y* yes, *N* no

^a^Sample size refers to number of legs, not the number of subjects, in the trial

^b^Included in meta-analysis by Shang et al. [http://www.ispm.unibe.ch/unibe/portal/fak_medizin/ber_vkhum/inst_smp/content/e93945/e93964/e180045/e180897/1433.Study_characteristics_of_homoeopathy_studies_corrected_eng.pdf (accessed 1 July 2016)]

### Risk of bias and reliable evidence

Table 3 provides the risk-of-bias details for each of the 75 trials, and sub-divided by: (a) the 54 that could be included in meta-analysis; (b) the 21 that could not be included in meta-analysis. Domains IV (completeness of outcome data), V (selective outcome reporting) and VI (other sources of bias) presented the greatest methodological concerns. Sixteen of 30 trials that were *high risk of bias* for domain V were so because their data were not extractable for meta-analysis (see *Study selection for meta-analysis* above). Domain II (allocation concealment) presented the most uncertain methodological judgments, with 55 (73%) trials assessed *unclear risk of bias* and only 14 (19%) *low risk of bias*.

**Table 3:** Risk-of-bias assessments for trials: (a) included in meta-analysis; (b) not included in meta-analysis

			Risk-of-bias domain								
#	First author	Year	I	II	IIIa	IIIb	IV	V ^c^	VI	Risk of bias	Risk-of-bias rating
*(a) Included in meta-analysis*											
A272	Colau	2012	Y	Y	Y	Y	Y	Y	Y	Low ^a^	A
A103	Padilha	2011	Y	Y	Y	Y	Y	Y	Y	Low ^a^	A
A120	Singer	2010	Y	Y	Y	Y	Y	Y	U	Uncertain ^a^	B1*
A123	Taylor	2000	Y	U	Y	Y	Y	Y	Y	Uncertain	B1
A47	Baker	2003	Y	Y	Y	Y	U	Y	U	Uncertain	B2
A61	Cornu	2010	Y	Y	U	U	Y	Y	Y	Uncertain	B2
A67	Frass	2005	U	U	Y	Y	Y	Y	Y	Uncertain	B2
A93	Lewith	2002	U	U	Y	Y	Y	Y	Y	Uncertain	B2
A275	Naidoo	2013	U	U	Y	Y	Y	Y	Y	Uncertain	B2
A105	Paris	2008	U	Y	Y	Y	U	Y	Y	Uncertain	B2
A126	Tveiten	1998	Y	U	Y	Y	U	Y	Y	Uncertain	B2
A128	Vickers	1998	Y	Y	Y	Y	U	Y	U	Uncertain	B2
A137	Zabolotnyi	2007	Y	U	Y	Y	Y	U	Y	Uncertain	B2
A100	Oberbaum	2001	U	U	Y	Y	Y	Y	U	Uncertain	B3
A62	Diefenbach	1997	U	U	Y	U	U	Y	Y	Uncertain	B4
A64	Ferley	1989	U	U	Y	U	Y	U	Y	Uncertain	B4
A79	Hofmeyr	1990	Y	U	U	U	Y	U	Y	Uncertain	B4
A92	Leaman	1989	U	U	Y	Y	U	Y	U	Uncertain	B4
A293	Malapane	2014	U	U	U	U	Y	Y	Y	Uncertain	B4
A125	Tveiten	1991	U	U	Y	U	U	Y	Y	Uncertain	B4
A135	Wiesenauer	1995	U	U	Y	Y	U	Y	U	Uncertain	B4
A112	Reilly	1994	U	U	U	U	Y	Y	U	Uncertain	B5
A48	Balzarini	2000	U	U	U	U	U	Y	U	Uncertain	B6
A75	GRECHO	1989	U	U	U	U	U	Y	U	Uncertain	B6
A83	Kaziro	1984	U	U	U	U	U	Y	U	Uncertain	B6
A104	Papp	1998	U	U	Y	U	U	U	U	Uncertain	B6
A81	Jacobs	2007	Y	Y	Y	Y	Y	Y	N	High	C1.0
A131	Wiesenauer	1985	Y	U	Y	Y	N	Y	Y	High	C1.1
A68	Freitas	1995	Y	U	Y	Y	N	Y	U	High	C1.2
A111	Reilly	1986	U	U	Y	Y	N	Y	Y	High	C1.2
A113	Robertson	2007	Y	U	Y	Y	N	Y	U	High	C1.2
A133	Wiesenauer	1990	U	U	Y	Y	N	Y	Y	High	C1.2
A86	Kim	2005	Y	U	U	U	N	Y	Y	High	C1.3
A134	Wiesenauer	1991	U	U	Y	U	N	Y	Y	High	C1.3
A59	Cialdella	2001	U	U	U	U	N	Y	Y	High	C1.4
A63	Ernst	1990	U	U	U	U	Y	Y	N	High	C1.4
A56	Bignamini	1987	U	U	U	U	U	Y	N	High	C1.5
A84	Khuda-Bukhsh	2005	U	U	U	U	U	U	N	High	C1.6
A94	Lipman	1999	U	Y	Y	Y	N	N	Y	High	C2.1
A108	Rahlfs	1976	N	N	Y	Y	U	Y	Y	High	C2.1
A70	Friese	1997	U	U	Y	Y	N	N	Y	High	C2.2
A74	Gerhard (a)	1998	Y	U	Y	U	N	Y	N	High	C2.2
A74	Gerhard (b)	1998	Y	U	Y	U	N	Y	N	High	C2.2
A74	Gerhard (c)	1998	Y	U	Y	U	N	Y	N	High	C2.2
A89	Kotlus	2010	N	N	U	U	Y	Y	Y	High	C2.2
A50	Belon	2006	N	N	U	U	Y	Y	U	High	C2.3
A136	Wolf	2003	Y	U	U	U	Y	N	N	High	C2.3
A49	Beer	1999	U	U	U	U	Y	N	N	High	C2.4
A52	Bergmann (a)	2000	U	U	Y	U	U	N	N	High	C2.4
A52	Bergmann (b)	2000	U	U	Y	U	U	N	N	High	C2.4
A101	Oberbaum	2005	U	U	U	U	U	N	N	High	C2.5
A60	Clark	2000	U	U	U	U	N	N	N	High	C3.4
A85	Khuda-Bukhsh	2011	N	N	Y	Y	N	N	N	High	C5.0
A109	Rahlfs	1978	N	N	N ^d^	N	N	Y	Y	High	C5.0
*(b) Not included in meta-analysis*											
A80	Jacobs	2006	Y	Y	Y	Y	Y	N ^b^	Y	High ^f^	C1.0
A91	Labrecque	1992	Y	U	Y	Y	U	N ^b^	Y	High ^e^	C1.2
A274	Harrison	2013	Y	U	Y	Y	U	N ^b^	U	High ^e^	C1.3
A78	Hitzenberger	2005	U	U	Y	U	U	N ^b^	Y	High ^e^	C1.4
A277	Razlog	2012	U	U	Y	Y	U	N ^b^	U	High ^e^	C1.4
A117	Seeley	2006	U	U	U	U	Y	N ^b^	U	High ^e^	C1.5
A55	Berrebi	2001	U	U	U	U	U	N ^b^	U	High ^e^	C1.6
A76	Hart	1997	Y	Y	Y	Y	U	N	Y	High	C1.1
A116	Schmidt	2002	Y	Y	Y	Y	Y	N ^b^	N	High	C2.0
A122	Stevinson	2003	Y	Y	Y	Y	Y	N ^b^	N	High	C2.0
A278	Sencer	2012	U	Y	Y	Y	N	N ^b^	Y	High	C2.1
A130	Weiser	1994	Y	U	Y	Y	N	N	Y	High	C2.1
A43	Aabel	2000	U	U	Y	Y	Y	N ^b^	N	High	C2.2
A44	Aabel	2000	U	U	Y	Y	N	N ^b^	U	High	C2.2
A42	Aabel	2001	U	U	U	Y	Y	N ^b^	N	High	C2.3
A132	Wiesenauer	1989	U	U	Y	U	N	N	Y	High	C2.3
A53	Bernstein	2006	U	U	U	U	U	N ^b^	N	High	C2.5
A69	Friese	2007	U	U	U	U	N	N	N	High	C3.4
A88	Kolia-Adam	2008	N	U	U	U	U	N	N	High	C3.4
A95	McCutcheon	1996	U	U	U	U	N	N ^b^	N	High	C3.4
A51	Belon	2007	N	N	N ^d^	N	N	N ^b^	U	High	C6.1

Trials are arranged by risk of bias per category (a) and (b)

*Y* yes (low risk of bias), *U* unclear, *N* no (high risk of bias)

^a^Reliable evidence

^b^Data not extractable for meta-analysis

^c^Unless a published study protocol was available, completeness of reporting was judged solely on correspondence of Results with details in Methods section of paper

^d^A51 Belon and A109 Rahlfs, on initial full-text scanning, were deemed to have satisfactory participant/practitioner blinding – later refuted in detailed scrutiny

^e^Except for domain V (data not extractable for meta-analysis), trial is otherwise *uncertain risk of bias* overall

^f^Except for domain V (data not extractable for meta-analysis), trial is otherwise *low risk of bias* overall

There were three trials with *reliable evidence* (two ‘A’-rated, one ‘B1*’-rated), 23 with *uncertain risk of bias* (‘B’-rated), and 49 with *high risk of bias* (‘C’-rated). A summary risk-of-bias bar-graph is shown in Additional file 3.

Table 3a *(54 trials included in meta-analysis):* Two trials were ‘A’-rated (low risk of bias)—i.e. they fulfilled the criteria for all seven domains of assessment. Our criteria for reliable evidence were also satisfied for one ‘B1*’-rated trial. Table 3a therefore includes three trials that were classed *reliable evidence*: *Plumbum metallicum* for lead poisoning (A103: Padilha); the OTC complex *Acthéane* for menopausal syndrome (A272: Colau); the OTC complex *Traumeel S* for post-operative pain (A120: Singer). Each of the other 51 trials had uncertain or high risk of bias in important methodological aspects, and may be regarded as *non-reliable evidence:* 23 trials were classed as *uncertain risk of bias*; 28 were classed as *high risk of bias*.

Table 3b *(21 trials excluded from meta-analysis):* All of these 21 trials are ‘C’-rated (high risk of bias). Thirteen of the 21 were seriously flawed in more than one domain of assessment (i.e. rated ‘C2.0’ or worse). Seven of the remaining eight trials were ‘C’-rated solely because of data extraction issues: only one of those seven (A80: Jacobs) fulfilled ‘low risk-of-bias’ criteria for all other domains of assessment, and so would otherwise have been designated *reliable evidence*.

### Meta-analysis

**Fig. 2:**
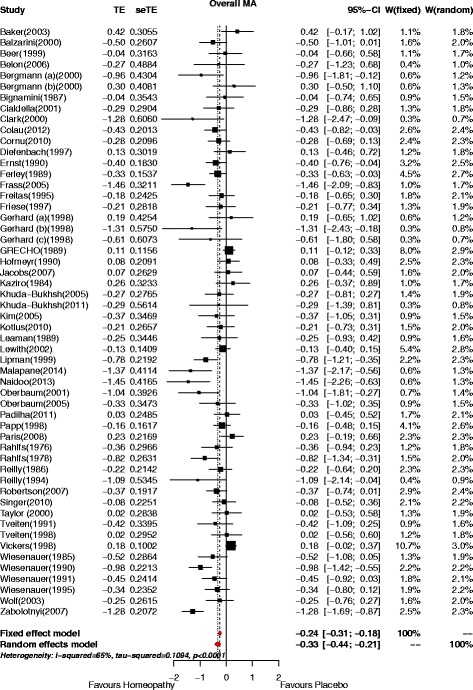
Forest plot for 54 analysable RCTs of non-individualised homeopathy. Shows SMD (Treatment Effect, TE) and 95% confidence interval (CI). Pooled effects estimate shown for fixed-effect and random-effects model. *W* weighting

The pooled SMD (random-effects model) for all 54 trials was –0.33 (95% CI –0.44, –0.21; *p* < 0.001)—see Fig. 2.

The original data extracted per trial (continuous or dichotomous), together with the correspondingly calculated SMD or OR, are illustrated in Additional files 4a and b.

Of the 31 trials with *continuous data*, 9 had an effect statistically significantly favouring homeopathy (i.e. SMD < 0, with *p* ≤ 0.05); no trials had an effect significantly favouring placebo. The pooled effect estimate was SMD = –0.36 (95% CI –0.52, –0.19; *p* < 0.001). Of the 23 trials with *dichotomous data*, 6 had an effect statistically significantly favouring homeopathy (i.e. OR > 1, with *p* ≤ 0.05); no trials had an effect significantly favouring placebo. The pooled effect estimate was OR = 1.67 (95% CI 1.25, 2.23; *p* < 0.001).

#### Heterogeneity and publication bias

The statistical heterogeneity among the studies was high (*I* ^2^ = 65%) – Fig. 2.

**Fig. 3:**
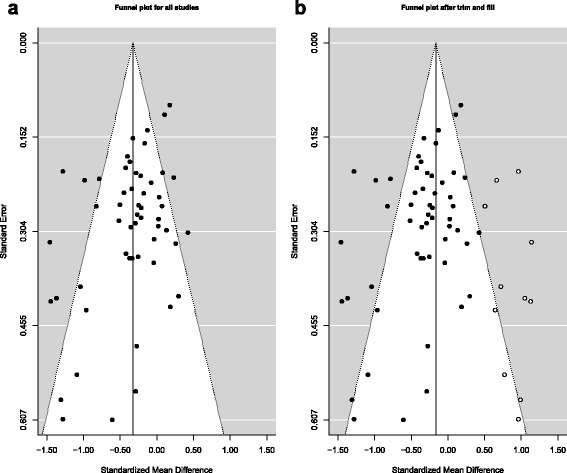
**a** Funnel plot for 54 RCTs of non-individualised homeopathy. Central *vertical line* is pooled effect estimate: SMD = –0.33. Heterogeneity statistic (*I* ^2^) = 65%. **b** Funnel plot for 54 RCTs of non-individualised homeopathy after ‘trim and fill’. Central vertical line is pooled effect estimate: SMD = –0.16. Heterogeneity statistic (*I* ^2^ ) = 79%

Evidence of publication bias, toward studies favouring homeopathy, was apparent from the funnel plot (Fig. 3a), which suggested a relative absence of studies favouring placebo. Egger’s test of asymmetry confirmed significant evidence of asymmetry in the funnel plot, *p* = 0.002. The estimated number of ‘missing’ studies was 11 (*p* for at least one ‘missing’ study was <0.001) – Fig. 3b. The effect estimate was attenuated when using the ‘trim-and-fill’ method to adjust for publication bias: after adjustment for ‘missing’ studies, the pooled effect estimate was –0.16 (95% CI –0.31, –0.02; *p* = 0.023); the statistical heterogeneity among the studies remained high (*I* ^2^ = 79%).

#### Risk of bias and reliable evidence

**Fig. 4:**
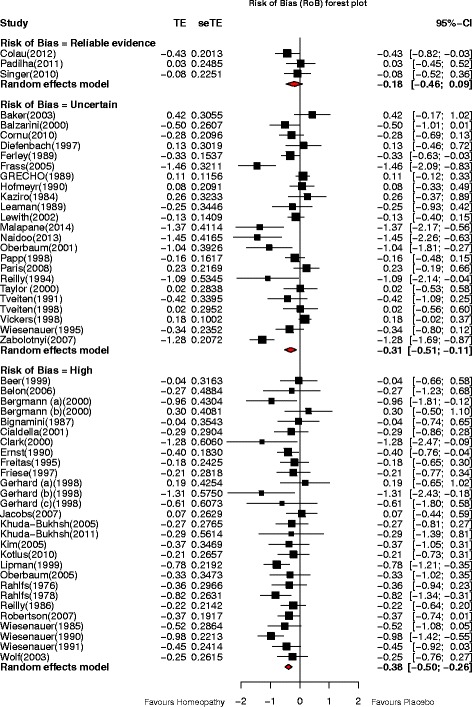
Forest plots showing SMD (Treatment Effect, TE) and 95% confidence interval (CI) for RCTs of non-individualised homeopathy, with pooled SMD (random-effects model) for trials assessed as *minimal or low risk of bias* (*reliable evidence*; *N* = 3); *uncertain risk of bias* (*non-reliable evidence*; *N* = 23); *high risk of bias* (*non-reliable evidence*; *N* = 28)

Figure 4 shows the SMD data for all 54 analysable trials, grouped by their risk of bias (high; uncertain; minimal or low [reliable evidence]).

- *High risk of bias/non-reliable evidence* (‘C’-rated: *N* = 28): SMD = –0.38 (95% CI –0.50, –0.26; *p* < 0.001);
- *Uncertain risk of bias/non-reliable evidence* (‘B’-rated: *N* = 23): SMD = –0.31 (95% CI –0.51, –0.11; *p* = 0.002);
- *Minimal or low risk of bias/reliable evidence* (‘B1*’ plus ‘A’-rated: *N* = 3): SMD = –0.18 (95% CI –0.46, 0.09; *p* = 0.165).

From this risk-of-bias analysis, no significant difference was detected between the three pooled effect estimates (*p* = 0.417); meta-regression confirmed this finding (*p* = 0.617). There was thus no statistical evidence that effect estimates significantly differed depending on whether the body of evidence for a meta-analysis consisted of ‘low’, ‘uncertain’ or ‘high’ risk-of-bias studies.

### Sensitivity analysis

**Fig. 5:**
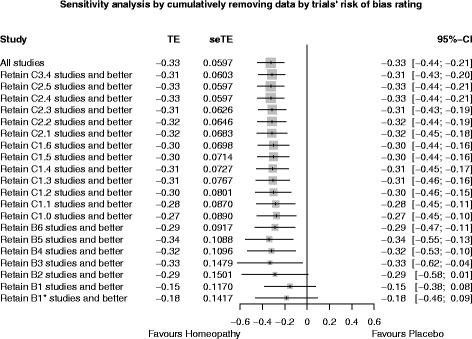
Sensitivity analysis, showing progressive effect on pooled SMD (*treatment effect* TE) of removing data by trials’ risk-of-bias rating

Figure 5 shows the effect of cumulatively removing data by trials’ risk-of-bias rating. The pooled SMD showed a statistically significant effect in favour of homeopathy for all trials collectively, through to and including those rated ‘B3’; for the highest-rated trials collectively (‘B2’, ‘B1’ and ‘reliable evidence’), the pooled SMD still favoured homeopathy but was no longer statistically significant.

### Sub-group analyses

**Fig. 6:**
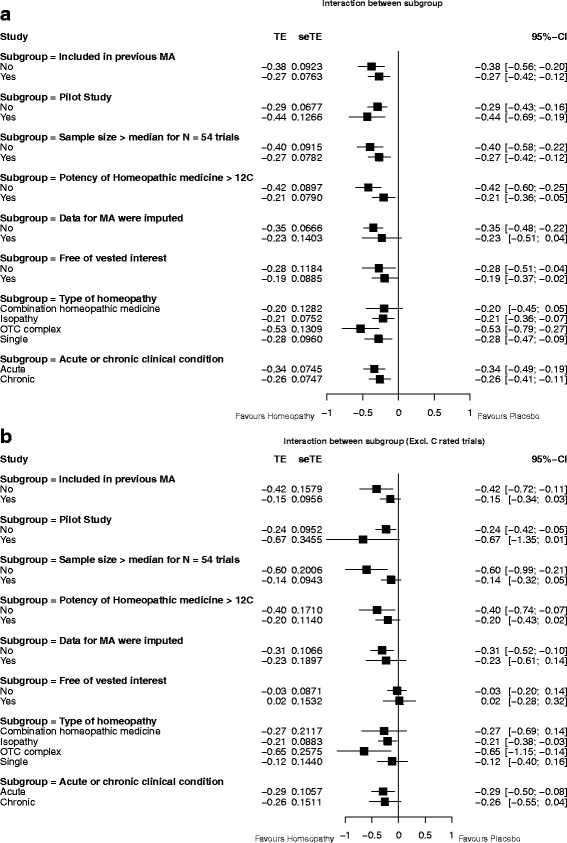
Interactions between sub-groups for: **a** all *N* = 54 trials with analysable data; **b** *N* = 26 ‘A’- and ‘B’-rated trials

The pooled SMD favoured homeopathy for all sub-groups, though it was statistically non-significant for two of the 18 (data imputed; combination medicine): Fig. 6a. A meta-regression was performed to test specifically for within-group differences for each sub-group. The results showed that there were no significant differences between studies that were and were not: included in previous meta-analyses (*p* = 0.447); pilot studies (*p* = 0.316); greater than the median sample (*p* = 0.298); potency ≥ 12C (*p* = 0.221); imputed for meta-analysis (*p* = 0.384); free from vested interest (*p* = 0.391); acute/chronic (*p* = 0.796); different types of homeopathy (*p* = 0.217).

After removal of ‘C’-rated trials (Fig. 6b), the pooled SMD still favoured homeopathy for all sub-groups, but was statistically non-significant for 10 of the 18 (included in previous meta-analysis; pilot study; sample size > median; potency ≥12C; data imputed; free of vested interest; not free of vested interest; combination medicine; single medicine; chronic condition). There remained no significant differences *between sub-groups*—with the exception of the analysis for sample size > median (*p* = 0.028).

#### Analysis by clinical condition

##### Clinical conditions

**Fig. 7:**
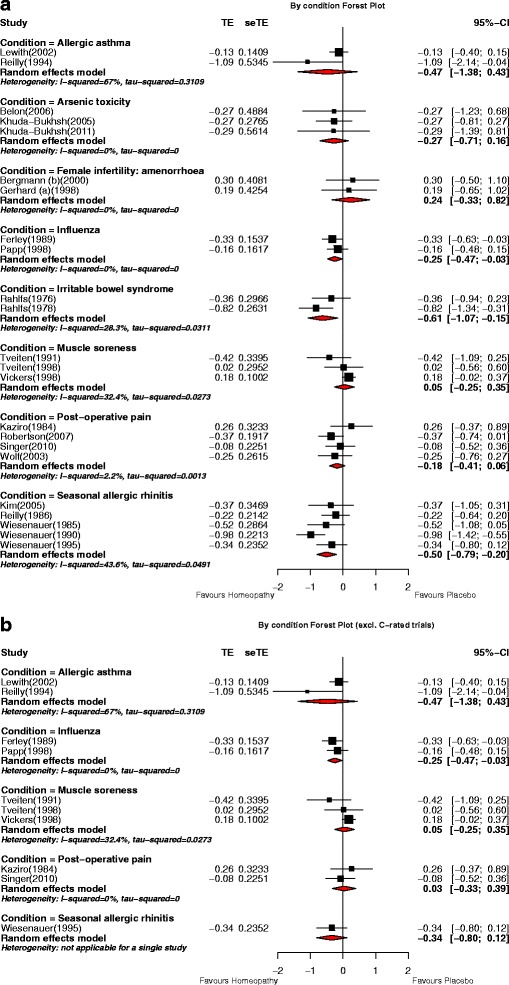
Meta-analysis by clinical condition for: **a** all *N* = 54 trials with analysable data; **b** *N* = 26 ‘A’- and ‘B’-rated trials. *p* values for pooled effect estimates: **a** Allergic asthma: *p* = 0.307; arsenic toxicity: *p* = 0.219; female infertility (amenorrhoea): *p* = 0.407; influenza: *p* = 0.025; irritable bowel syndrome: *p* = 0.009; muscle soreness: *p* = 0.762; post-operative pain: *p* = 0.143; seasonal allergic rhinitis: *p* = 0.001. **b** Allergic asthma: *p* = 0.307; influenza: *p* = 0.025; muscle soreness: *p* = 0.762; post-operative pain: *p* = 0.859; seasonal allergic rhinitis: *p* = 0.147

Meta-analysis was possible for eight clinical conditions, each analysis comprising two to five trials (Fig. 7a). A statistically significant pooled SMD, favouring homeopathy, was observed for influenza (*N* = 2), irritable bowel syndrome (*N* = 2), and seasonal allergic rhinitis (*N* = 5). Each of the other five clinical conditions (allergic asthma, arsenic toxicity, infertility due to amenorrhoea, muscle soreness, post-operative pain) showed non-significant findings. Removal of ‘C’-rated trials negated the statistically significant effect for seasonal allergic rhinitis and left the non-significant effect for post-operative pain unchanged (Fig. 7b); no higher-rated trials were available for additional analysis of arsenic toxicity, infertility due to amenorrhoea or irritable bowel syndrome. There were no ‘C’-rated trials to remove for allergic asthma, influenza, or muscle soreness. Thus, influenza was the only clinical condition for which higher-rated trials indicated a statistically significant effect; neither of its contributing trials, however, comprised reliable evidence.

#### Categories of clinical condition

**Fig. 8:**
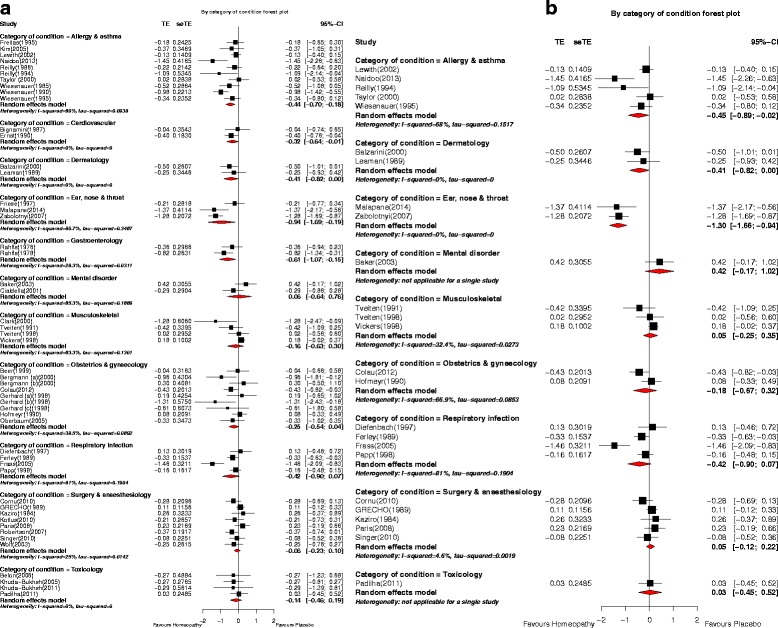
Meta-analysis by category of clinical condition for: **a** all *N* = 54 trials with analysable data; **b** *N* = 26 ‘A’- and ‘B’-rated trials. *p* values for pooled effect estimates: **a** Allergy and asthma: *p* = 0.001; Cardiovascular: *p* = 0.046; Dermatology: *p* = 0.047; Ear, nose and throat: *p* = 0.014; Gastroenterology: *p* = 0.009; Mental disorder: *p* = 0.865; Musculoskeletal: *p* = 0.488; Obstetrics and gynaecology: *p* = 0.088; Respiratory infection: *p* = 0.092; Surgery and anaesthesiology: *p* = 0.448; Toxicology: *p* = 0.406. **b** Allergy and asthma: *p* = 0.041; Dermatology: *p* = 0.047; Ear, nose and throat: *p* < 0.001; Mental disorder: *p* = 0.165; Musculoskeletal: *p* = 0.762; Obstetrics and gynaecology: *p* = 0.486; Respiratory infection: *p* = 0.092; Surgery and anaesthesiology: *p* = 0.576; Toxicology: *p* = 0.896

Meta-analysis was possible for 11 categories of clinical condition, each analysis comprising two to ten trials (Fig. 8a). A statistically significant pooled SMD, favouring homeopathy, was observed for five categories: allergy and asthma (*N =* 10); cardiovascular (*N =* 2); dermatology (*N =* 2); ear nose and throat (*N =* 3); gastroenterology (*N =* 2). None of the trials designated reliable evidence featured in any of these five categories. Each of the other six categories showed non-significant findings. Removal of ‘C’-rated trials limited each analysis to two to five trials (Fig. 8b): statistically significant effects were marginally retained for allergy and asthma (*N =* 5) and dermatology (*N =* 2), and more clearly retained for ear nose and throat (*N =* 2). No higher-rated trials were available for additional analysis in the cardiovascular and gastroenterology categories. After removal of ‘C’-rated trials, there was no change in the non-significance of the statistical findings for each of the other six categories.

## Discussion

Seventy-two of the 75 eligible trials had uncertain or high risk of bias. Due to poor reporting or other deficiencies in 21 of the original papers, data extraction for our meta-analysis was possible from only 54 of the 75 trials. Trials with high and with uncertain risk of bias each featured similarly in our 54-trial analysis; the quality of the body of analysed evidence is therefore low.

As previously recognised [2, 7, 9], the pooling of data from diverse clinical conditions, outcome measures and end-points has obvious limitations: thus, a given pooled effect estimate here does not have a clear numerical meaning or relative clinical value, but provides a reasonable summary measure in evaluating the average effect of a medical intervention. Our null hypothesis that regards each trial of non-individualised homeopathy as testing *the same* intervention also has its limitations, for it makes the debatable assumption that each homeopathic medicine has similar lack of efficacy for the relevant symptoms of every clinical condition. Nevertheless, our separate focus on individualised [2] and non-individualised homeopathy marks a clear and appropriate step forward.

For our previous meta-analysis of RCTs (on *individualised* homeopathy [2]), the three most highly ranked trials had minimal risk of bias and were designated reliable evidence. In the current study, we have identified two trials with the highest-quality ranking (‘A’ = low risk of bias), plus one with minimal risk of bias (‘B1*’), which we have examined collectively as the reliable evidence of RCTs of non-individualised homeopathic treatment. Analysis of these three highest-quality trials showed a statistically non-significant pooled SMD of –0.18 (95% CI –0.46, 0.09) (equivalent to pooled OR = 1.39, using the standard conversion [14]). This effect estimate of –0.18 contrasts with that for all 54 analysable trials of –0.33 (equivalent to OR = 1.82): the latter represents a small and statistically significant treatment effect favouring homeopathy, akin to our pooled findings for the individualised trials [2]. We therefore reject the null hypothesis (non-individualised homeopathy is indistinguishable from placebo) on the basis of pooling all studies, but fail to reject the null hypothesis on the basis of the reliable evidence only. Our risk-of-bias analysis and the meta-regression, however, indicate that effect estimates do not significantly differ depending on whether the meta-analysis consists of ‘low’, ‘uncertain’ or ‘high’ risk-of-bias studies.

Lack of clear conclusion above might simply be due to there being too few high-quality trials. With only three studies that can be classified as reliable evidence, it is difficult to separate an effect of homeopathy from the effect of poor quality. The three studies comprising ‘reliable’ RCT evidence are clinically heterogeneous: *Plumbum metallicum* for lead poisoning ([23]; null effect); *Acthéane* for menopausal syndrome ([24]; significant treatment effect; evidence of vested interest); *Traumeel S* for post-operative pain ([25]; null effect). Since the completion of our defined literature search, we are aware of recently published and potentially eligible RCT papers, whose findings we have yet to explore [26–29]. The limit of detecting an effect of non-individualised homeopathy across all trials may be related to a medicine’s degree of dilution, since trials using potency ≥12C failed to show a statistically significant pooled effect that favoured homeopathy (see Fig. 6b).

In attempting to formulate a reasonable overarching conclusion, it is important also to highlight other findings from our quality-based analyses. For example, the sensitivity analysis that consecutively excluded the lowest-quality trials showed that studies with lower quality tended to report greater benefits of non-individualised homeopathic intervention than studies with higher quality. That RCTs with a higher risk of bias showed a greater benefit for the homeopathy group supports some previous—though not our own [2]—meta-analysis findings [4, 7, 10]. Our funnel plot finding of larger effect estimates (in favour of homeopathy) in trials with lower sample size is consistent with observations from RCTs in medicine more widely [30]. A further perspective, based on our trim-and-fill analysis, is that the true pooled effect estimate is likely to be smaller than initially appreciated: we found evidence of publication bias, with an estimated 11 ‘missing’ studies whose results would favour placebo, adjustment for which yielded an attenuated but still-significant pooled effect estimate of –0.16 for the 54 analysable trials. We are also aware that our analysis reflects per-protocol—not the potentially more robust (but less available) ITT—outcome data, which might have slightly magnified our pooled effect estimate; however, we have addressed the possible impact of incomplete data in rigorous risk-of-bias assessments, as recommended by Cochrane [31]. The sum of these comments supports a generalised conclusion that a non-individualised homeopathic medicine is indistinguishable from a placebo, but the quality of the evidence is low.

A small and erratic treatment effect in this context may be consistent with the notion that a pre-selected homeopathic medicine, aiming to treat the typical symptoms of a clinical condition, and given to *all* of the relevant trial participants, may match sub-optimally the ‘total symptom picture’ for an important number of them, leading potentially to diminished efficacy. The quality of the clinical intervention and the suitability of the main outcome measure are the key facets of a trial’s *model validity*, i.e. the extent to which a study reflects best clinical practice in that intervention [32]. Thus, to complete the quality evaluation of homeopathy trials, it is important to accommodate also the assessment of their model validity, emphasising in this case the three trials comprising reliable evidence in non-individualised homeopathic treatment.

We report separately our model validity assessments of these trials, evaluating consequently their overall quality based on a *GRADE*-like principle of ‘downgrading’ [14]: two trials [23, 25] rated here as reliable evidence were downgraded to ‘low quality’ overall due to the inadequacy of their model validity; the remaining trial with reliable evidence [24] was judged to have adequate model validity. The latter study [24] thus comprises the sole RCT that can be designated ‘high quality’ overall by our approach^5^, a stark finding that reveals further important aspects of the preponderantly low quality of the current body of evidence in non-individualised homeopathy.

Analysis by clinical condition, and following removal of ‘C’-rated studies, showed a statistically significant treatment effect in RCTs of non-individualised homeopathy for influenza, and in the categories allergy and asthma, dermatology, and ear nose and throat. None of these analyses included any reliable evidence, however. While these clinical categories do not provide compelling evidence for non-individualised homeopathic treatment, they may contain the most promising targets for future research.

## Conclusions

There was a small, statistically significant, effect of non-individualised homeopathic treatment. However, the finding was not robust to sensitivity analysis based solely on the three trials that comprised reliable evidence: the effect size estimate collectively for those three trials was not statistically significant. There was significant evidence of publication bias in favour of homeopathy. Our meta-analysis of the current reliable evidence base therefore fails to reject the null hypothesis that the outcome of treatment using a non-individualised homeopathic medicine is not distinguishable from that using placebo. Nevertheless, the risk-of-bias analysis and the meta-regression, together with the large preponderance of low-quality evidence, challenge the inference that effect size estimates differ significantly depending on risk-of-bias rating. The assessment of a trial’s model validity should also be taken into account in an evaluation of overall study quality in homeopathy. Reliable evidence is lacking for all clinical conditions whose data have enabled separate meta-analysis. Higher-quality RCT research on specified homeopathic medicines is required to enable more decisive interpretation regarding efficacy for given clinical symptoms or conditions. Future trialists need to minimise their studies’ risk of bias in all domains, and to improve the clarity of their reporting. Such research might wisely focus on trial design in which only patients that match the relevant ‘symptom picture’ or match the indications of the selected homeopathic product are those eligible to participate: large trials are therefore indicated.

## Additional files

Additional file 1: Checklist. *PRISMA* 2009 Checklist. (DOC 66 kb)

Additional file 2: Details of records of non-individualised homeopathy included in, and excluded from, systematic review and meta-analysis. SD, standard deviation. In comparison to the protocol [3], A110 Ramelet has been excluded from this systematic review due to its updated identification as a *prophylaxis* trial. (DOCX 76 kb)

Additional file: 3 Risk-of-bias bar-graph for 75 RCTs of non-individualised homeopathy. (DOCX 170 kb)

Additional file 4: Forest plots, showing (a) standardised mean difference (SMD) and (b) odds ratio (OR), with 95% confidence interval (CI) for original data (continuous or dichotomous) extracted per trial of non-individualised homeopathy. Pooled effects estimate shown for fixed-effect and random-effects model. W, weighting. To ensure consistent direction of measurement with disease severity, sign inversion was applied to the mean value of five trials in (a). [Reflecting the fact that OR > 1 favours homeopathy, the direction of change toward homeopathy in plot (b) is to the *right*, thus differing from all other plots]. (ZIP 15 kb)

## Abbreviations

CI: Confidence interval
*GRADE*: Grades of Recommendation, Assessment, Development and Evaluation
ICF: International Classification of Functioning
ITT: Intention to treat
OR: Odds ratio
OTC: Over-the-counter
*PRISMA*: Preferred Reporting Items for Systematic Reviews and Meta-Analyses
RCT: Randomised controlled trial
SD: Standard deviation
SMD: Standardised mean difference
WHO: World Health Organization

## Footnotes

1 In practice, data were not derivable from the three relevant trials with continuous data: due to the original authors’ use of either medians or ‘change’ data only.

2 This nomenclature is not intended to be definitive, but a basis for more refined analysis at a later date.

3 Complete details of all 553 records are available at: https://www.hri-research.org/hri-research/learning-more-from-existing-evidence/systematic-review-programme/

4 Additional file 2 represents an update of the flowchart included in the original study protocol [3].

5 Mathie RT, Van Wassenhoven M, Rutten ALB, Klein-Laansma CT, Eizayaga J, Pla i Castellsagué A, Jong MC, Manchanda RK, Dantas F, Oberbaum M, Frye J, Roniger H, Baumgartner S, van Haselen R, Nicolai T, Fisher P. Model validity of randomised placebo-controlled trials of non-individualised homeopathic treatment. Submitted for publication.
