# Supplementary material for: Randomised, double-blind, placebo-controlled trials of non-individualised homeopathic treatment: systematic review and meta-analysis
Source: Syst Rev. 2017 Mar 24;6:63. doi: 10.1186/s13643-017-0445-3 (PMC5366148; doi:10.1186/s13643-017-0445-3)
Supplement: Supplementary file 2 — Details of records of non-individualised homeopathy included in, and excluded from, systematic review and meta-analysis. SD, standard deviation. In comparison to the protocol [3], A110 Ramelet has been excluded from this systematic review due to its updated identification as a prophylaxis trial. (DOCX 76 kb) [file 13643_2017_445_MOESM2_ESM.docx]

**Additional File 2**

**38 records excluded from systematic review**:

***Prophylaxis****:* ***Single-blinded****:*

A58: Brydak A73: Garrett

A98: Mokkapatti A99: Mousavi

A110: Ramelet A294: Patil

A276: Pérol

A295: Nair ***Lab. Experiment****:*

A82: Jawara

***Crossover****:* A96: Meissner

A46: Baillargeon A106: Paris

A66: Fisher A107: Plezbert

A77: Heusser A124: Tuten

A90: La Pine A127: Vickers

A114: Saruggia A269: Bell

A118: Shipley A271: Bell

A119: Simpson

A121: Smith ***Other****:*

A129: von Hagens A45: Adkison

A273: Dean A57: Brinkhaus

A65: Ferrara

***Combined therapy****:* A87: Kneis

A54: Bernstein A97: Merklinger

A71: Furuta A102: Pach

A72: Furuta A270: Bell

A115: Schirmer A279: Zanasi

**14 records (as per search updates for 2012-2013, 2014)**:

**A269–A279, A293–A295**

**96 records (as per original**

**literature search)**:

**A42–A137**

**72 records included in systematic review**

**51 records included in meta-analysis**:

A47: Baker A79: Hofmeyr A111: Reilly

A48: Balzarini A81: Jacobs A112: Reilly

A49: Beer A83: Kaziro A113: Robertson

A50: Belon A84: Khuda-Bukhsh A120: Singer

A52: Bergmann A85: Khuda-Bukhsh A123: Taylor

A56: Bignamini A86: Kim A125: Tveiten

A59: Cialdella A89: Kotlus A126: Tveiten

A60: Clark A92: Leaman A128: Vickers

A61: Cornu A93: Lewith A131: Wiesenauer

A62: Diefenbach A94: Lipman A133: Wiesenauer

A63: Ernst A100: Oberbaum A134: Wiesenauer

A64: Ferley A101: Oberbaum A135: Wiesenauer

A67: Frass A103: Padilha A136: Wolf

A68: Freitas A104: Papp A137: Zabolotnyi

A70: Friese A105: Paris A272: Colau

A74: Gerhard A108: Rahlfs A275: Naidoo

A75: GRECHO A109: Rahlfs A293: Malapane

**21 records excluded from meta-analysis**:

***Data not provided****:* ***SDs not derivable****:*

A69: Friese A51: Belon

A76: Hart A53: Bernstein

A91: Labrecque A55: Berrebi

A278: Sencer A78: Hitzenberger

A88: Kolia-Adam

***Non-parametric data****:* A95: McCutcheon

A42: Aabel A116: Schmidt

A43: Aabel A117: Seeley

A44: Aabel A130: Weiser

A80: Jacobs A274: Harrison

A122: Stevinson A277: Razlog

A132: Wiesenauer

A60: Clark

**References for Additional file 4**:

(Red font: Ineligible for current systematic review)

| A42 | Aabel S (2001). Prophylactic and acute treatment with the homeopathic medicine Betula 30c for birch pollen allergy: a double-blind, randomized, placebo-controlled study of consistency of VAS responses. British Homeopathic Journal; 90:73–78. |
| --- | --- |
| A43 | Aabel S, Laerum E, Dølvik S, Djupesland P (2000). Is homeopathic 'immunotherapy' effective? A double-blind, placebo-controlled trial with the isopathic remedy Betula 30c for patients with birch pollen allergy. British Homeopathic Journal; 89:161–168. |
| A44 | Aabel S (2000). No beneficial effect of isopathic prophylactic treatment for birch pollen allergy during a low-pollen season: a double-blind, placebo-controlled clinical trial of homeopathic Betula 30c. British Homeopathic Journal; 89:169–173. |
| A45 | Adkison JD, Bauer DW, Chang T (2010). The effect of topical arnica on muscle pain. Annals of Pharmacotherapy; 44:1579–1584. |
| A46 | Baillargeon L, Drouin J, Desjardins L, Leroux D, Audet D (1993). Les effets de l'Arnica Montana sur la coagulation sanguine. Essai clinique randomisé [The effects of Arnica Montana on blood coagulation. Randomized controlled trial]. Canadian Family Physician; 39:2362–2367. |
| A47 | Baker DG, Myers SP, Howden I, Brooks L (2003). The effects of homeopathic Argentum nitricum on test anxiety. Complementary Therapies in Medicine; 11:65–71. |
| A48 | Balzarini A, Felisi E, Martini A, De Conno F (2000). Efficacy of homeopathic treatment of skin reactions during radiotherapy for breast cancer: a randomized, double-blind clinical trial. British Homeopathic Journal; 89:8–12. |
| A49 | Beer AM, Heiliger F (1999). Caulophyllum D4 zur Geburtsinduktion bei vorzeitigem Blasensprung - eine Doppelblindstudie [Randomized, double-blind trial of Caulophyllum D4 for induction of labour after premature rupture of the membranes at term]. Geburtshilfe und Frauenheilkunde; 59:431–435. |
| A50 | Belon P, Banerjee P, Choudhury SC, Banerjee A, Biswas SJ, Karmakar SR, Pathak S, Guha B, Chatterjee S, Bhattacharjee N, Das JK, Khuda-Bukhsh AR (2006). Can administration of potentized homeopathic remedy, Arsenicum album, alter antinuclear antibody (ANA) titre in people living in high-risk arsenic contaminated areas? I. A correlation with certain hematological parameters. Evidence-Based Complementary and Alternative Medicine; 3:99-107. |
| A51 | Belon P, Banerjee A, Karmakar SR, Biswas SJ, Choudhury SC, Banerjee P, Das JK, Pathak S, Guha B, Paul S, Bhattacharjee N, Khuda-Bukhsh AR (2007). Homeopathic remedy for arsenic toxicity? Evidence-based findings from a randomized placebo-controlled double blind human trial. Science of the Total Environment; 384:141–150. |
| A52 | Bergmann J, Luft B, Boehmann S, Runnebaum B, Gerhard I (2000). Die Wirksamkeit des Komplexmittels Phyto-Hypophyson® L bei weiblicher, hormonell bedingter Sterilität. Eine randomisierte, plazebokontrollierte, klinische Doppelblindstudie [The efficacy of the complex medication Phyto-Hypophyson L in female, hormone-related sterility. A randomized, placebo-controlled clinical double-blind study]. Forschende Komplementärmedizin und Klassische Naturheilkunde; 7:190–199. |
| A53 | Bernstein S, Donsky H, Gulliver W, Hamilton D, Nobel S, Norman R (2006). Treatment of mild to moderate psoriasis with Reliéva, a Mahonia aquifolium extract - a double-blind, placebo-controlled study. American Journal of Therapeutics; 13:121–126. |
| A54 | Bernstein JA, Davis BP, Picard JK, Cooper JP, Zheng S, Levin LS (2011). A randomized, double-blind, parallel trial comparing capsaicin nasal spray with placebo in subjects with a significant component of nonallergic rhinitis. Annals of Allergy, Asthma and Immunology; 107:171–178. |
| A55 | Berrebi A, Parant O, Ferval F, Thene M, Ayoubi JM, Connan L, Belon P (2001). Traitement de la douleur de la montée laiteuse non souhaitée par homéopathie dans le postpartum immédiat [Treatment of pain due to unwanted lactation with a homeopathic preparation given in the immediate post-partum period]. Journal de gynécologie, obstétrique et biologie de la reproduction; 30:353–357. |
| A56 | Bignamini M, Bertoli A, Consolandi AM, Dovera N, Saruggia M, Taino S, Tubertini A (1987). Controlled double-blind trial with Baryta carbonica 15CH versus placebo in a group of hypertensive subjects confined to bed in two old people's homes. British Homoeopathic Journal; 76:114–119. |
| A57 | Brinkhaus B, Wilkens JM, Lüdtke R, Hunger J, Witt CM, Willich SN (2006). Homeopathic arnica therapy in patients receiving knee surgery: results of three randomised double-blind trials. Complementary Therapies in Medicine; 14:237–246. |
| A58 | Brydak LB, Denys A (1999). The evaluation of humoral response and the clinical evaluation of a risk-group patients’ state of health after administration of the homeopathic preparation Gripp-Heel during the influenza epidemic season 1993/94. International Review of Allergology and Clinical Immunology; 5:223–227. |
| A59 | Cialdella P, Boissel JP, Belon P (2001). Spécialités homéopathiques en substitution de benzodiazépines: étude en double-insu vs. placebo [Complex homeopathic medicines as substitutes for benzodiazepines: double-blind study vs. placebo]. Thérapie; 56:397–402. |
| A60 | Clark J, Percivall A (2000). A preliminary investigation into the effectiveness of the homeopathic remedy, Ruta graveolens, in the treatment of pain in plantar fasciitis. British Journal of Podiatry; 3:81–85. |
| A61 | Cornu C, Joseph P, Gaillard S, Bauer C, Vedrinne C, Bissery A, Melot G, Bossard N, Belon P, Lehot J-J (2010). No effect of a homoeopathic combination of Arnica montana and Bryonia alba on bleeding, inflammation, and ischaemia after aortic valve surgery. British Journal of Clinical Pharmacology; 69:136-142. |
| A62 | Diefenbach M, Schilken J, Steiner G, Becker HJ (1997). Homöopathische Therapie bei Erkrankungen der Atemwege. Auswertung einer klinischen Studie bei 258 Patienten [Homeopathic therapy in respiratory tract diseases. Evaluation of a clinical study in 258 patients]. Zeitschrift für Allgemeinmedizin; 73:308–314. |
| A63 | Ernst E, Saradeth T, Resch KL (1990). Complementary therapy of varicose veins – a randomized, placebo-controlled, double-blind trial. Phlebology; 5:157–163. |
| A64 | Ferley JP, Zmirou D, D’Adhemar D, Balducci F (1989). A controlled evaluation of a homoeopathic preparation in the treatment of influenza like syndromes. British Journal of Clinical Pharmacology; 27:329–335. |
| A65 | Ferrara P, Marrone G, Emmanuele V, Nicoletti A, Mastrangelo A, Tiberi E, Ruggiero A, Fasano A, Paolini Paoletti F (2008). Homotoxicological remedies versus desmopressin versus placebo in the treatment of enuresis: a randomised, double-blind, controlled trial. Pediatric Nephrology; 23: 269-274. Epub 2007 Feb 20. |
| A66 | Fisher P, Greenwood A, Huskisson EC, Turner B, Belon P (1989). Effect of homoeopathic treatment on fibrositis (primary fibromyalgia). British Medical Journal; 299:365-366. |
| A67 | Frass M, Dielacher C, Linkesch M, Endler C, Muchitsch I, Schuster E, Kaye A (2005). Influence of potassium dichromate on tracheal secretions in critically ill patients. Chest; 127:936–941. |
| A68 | Freitas LAS, Goldenstein E, Sanna OM (1995). A relação médico-paciente indireta e o tratamento homeopático na asma infantile [The indirect patient-doctor relationship and the homeopathic treatment of childhood asthma]. Revista de Homeopatia; 60:26–31. |
| A69 | Friese K-H, Zabalotnyi DI (2007). Homöopathie bei akuter Rhinosinusitis. Eine doppelblinde, placebokontrollierte Studie belegt die Wirksamkeit und Verträglichkeit eines homöopathischen Kombinations-arzneimittels [Homeopathy in acute rhinosinusitis. A double-blind, placebo controlled study shows the efficiency and tolerability of a homeopathic combination remedy]. HNO; 55:271–277. |
| A70 | Friese K-H, Feuchter U, Möller H (1997). Die homöopathische Behandling von adenoiden Vegetationen [Homeopathic treatment of adenoid vegetations. Results of a prospective, randomized double-blind study]. HNO; 45:618–624. |
| A71 | Furuta SE, Weckx LLM, Figueiredo CR (2003). Estudo clínico, randomizado, duplocego, em crianças com adenóide obstrutiva, submetidas a tratamento homeopático [Prospective, randomized, double-blind clinical trial about efficacy of homeopathic treatment in children with obstructive adenoid]. Revista Brasileira de Otorrinolaringologia; 69:343–347. |
| A72 | Furuta SE, Weckx LLM, Figueiredo CR (2007). Tratamento Homeopático da amigdalite recorrente em crianças: um estudo randomizado controlado [Homeopathic treatment of recurrent tonsillitis in children: a randomized controlled trial]. Revista de Homeopatia; 70:21–26. |
| A73 | Garrett B, Harrison PV, Stewart T, Porter I (1997). A trial of homoeopathic treatment of leg ulcers. Journal of Dermatological Treatment; 8:115–117. |
| A74 | Gerhard I, Pateck A, Monga B, Blank A, Gorkow C (1998). Mastodynon bei weiblicher Sterilitat. Randomisierte, plazenbokontrollierte, klinische oppelblindstudie. [Mastodynon for female infertility. Randomised, placebo controlled, clinical double-blind study]. Forschende Komplementärmedizin; 5:272–278. |
| A75 | GRECHO (Groupe de Recherches et d'Essais Cliniques en Homéopathie), U292 INSERM, ARC (Association de Recherche en Chirurgie), GREPA (Groupe de Recherche et d’Étude de la Paroi Abdominal) (1989). Evaluation de deux produits homéopathiques sur la reprise du transit après chirurgie digestive – Un essai contrôlé multicentrique [Evaluation of the effects of two homeopathic preparations on the resumption of intestinal peristalsis after digestive tract surgery – A multicentre controlled trial]. Presse Médicale; 18:59–62. |
| A76 | Hart O, Mullee MA, Lewith G, Miller J (1997). Double-blind, placebo-controlled, randomized clinical trial of homoeopathic arnica C30 for pain and infection after total abdominal hysterectomy. Journal of the Royal Society of Medicine; 90:73–78. |
| A77 | Heusser P, Berger S, Stutz M, Hüsler A, Haeberli A, Wolf U (2009). Efficacy of homeopathically potentized antimony on blood coagulation. A randomized placebo controlled crossover trial. Forschende Komplementärmedizin; 16:14–18. |
| A78 | Hitzenberger G, Rehak PH (2005). Zur Wirkung eines homöopathi-schen Fertigarzneimittels auf den Blutdruck von Hypertonikern: Eine randomisierte doppelblinde kontrollierte Parallelgruppen-Vergleichsstudie [The effect of a homeopathic drug on the blood pressure of hypertensive patients: a randomized double-blind, controlled, parallel-group, comparative trial]. Wiener Medizinische Wochenschrift; 155:392–396. |
| A79 | Hofmeyr GJ, Piccioni V, Blauhof P (1990). Postpartum homoeopathic Arnica montana: a potency-finding pilot study. British Journal of Clinical Practice; 44:619–621. |
| A80 | Jacobs J, Guthrie BL, Montes GA, Jacobs LE, Mickey-Colman N, Wilson AR, DiGiacomo R (2006). Homeopathic combination remedy in the treatment of acute childhood diarrhea in Honduras. Journal of Alternative and Complementary Medicine; 12:723–732. |
| A81 | Jacobs J, Fernandez EA, Merizalde B, Avila-Montes GA, Crothers D (2007). The use of homeopathic combination remedy for dengue fever symptoms: a pilot RCT in Honduras. Homeopathy; 96:22–26. |
| A82 | Jawara N, Lewith G, Mullee M, Smith C (1997). Homoeopathic Arnica and Rhus Toxicodendron for delayed onset muscle soreness: a randomized, double-blind, placebo-controlled trial. British Homoeopathic Journal; 86:10–15. |
| A83 | Kaziro GS (1984). Metronidazole (Flagyl) and Arnica montana in the prevention of post-surgical complications, a comparative placebo controlled clinical trial. British Journal of Oral & Maxillofacial Surgery; 22:42–49. |
| A84 | Khuda-Bukhsh AR, Pathak S, Guha B, Karmakar SR, Das JK, Banerjee P, Biswas SJ, Mukherjee P, Bhattacharjee N, Choudhury SC, Banerjee A, Bhadra S, Mallick P, Chakrabarti J, Mandal B (2005). Can homeopathic arsenic remedy combat arsenic poisoning in humans exposed to groundwater arsenic contamination? A preliminary report on first human trial. Evidence-Based Complementary and Alternative Medicine; 2:537–548. |
| A85 | Khuda-Bukhsh AR, Banerjee A, Biswas SJ, Karmakar SR, Banerjee P, Pathak S, Guha B, Haque S, Das D, De A, Das D, Boujedaini N (2011a). An initial report on the efficacy of a millesimal potency Arsenicum Album LM 0/3 in ameliorating arsenic toxicity in humans living in a high-risk arsenic village. Journal of Chinese Integrative Medicine / Zhong Xi Yi Jie He Xue Bao; 9:596-604. |
| A86 | Kim LS, Riedlinger JE, Baldwin CM, Hilli L, Khalsa SV, Messer SA, Waters RF (2005). Treatment of seasonal allergic rhinitis using homeopathic preparation of common allergens in the southwest region of the US: a randomized, controlled clinical trial. Annals of Pharmacotherapy; 39:617–624. |
| A87 | Kneis KC, Gandjour A (2009). Economic evaluation of Sinfrontal® in the treatment of acute maxillary sinusitis in adults. Applied Health Economics and Health Policy; 7:181–191. |
| A88 | Kolia-Adam N, Solomon E, Bond J, Deroukakis M (2008). The efficacy of Coffea cruda on insomnia: a double blind trial. Simillimum; 21:91–99. |
| A89 | Kotlus BS, Heringer DM, Dryden RM (2010). Evaluation of homeopathic arnica montana for ecchymosis after upper blepharoplasty: A placebo-controlled, randomized, double-blind study. Ophthalmic Plastic and Reconstructive Surgery; 26:395–397. |
| A90 | La Pine MP, Malcomson FN, Torrance JM, Marsh NV (2006). Night shift: can a homeopathic remedy alleviate shift lag? Dimensions of Critical Care Nursing; 25:130–136. |
| A91 | Labrecque M, Audet D, Latulippe LG, Drouin J (1992). Homoeopathic treatment of plantar warts. Canadian Medical Association Journal; 146:1749–1753. |
| A92 | Leaman AM, Gorman D (1989). Cantharis in the early treatment of minor burns. Archives of Emergency Medicine; 6:259–261. |
| A93 | Lewith G, Watkins AD, Hyland ME (2002). Use of ultramolecular potencies of allergen to treat asthmatic people allergic to house dust mite: double blind randomized controlled clinical trial. British Medical Journal; 324:520–523. |
| A94 | Lipman D, Sexton G, Schlesser J (1999). A randomized double-blind placebo-controlled evaluation of the safety and efficacy of a natural over-the-counter (OTC) medication in the management of snoring. Sleep and Breathing; 3:53–56. |
| A95 | McCutcheon LE (1996). Treatment of anxiety with a homeopathic remedy. Journal of Applied Nutrition; 48:2–6. |
| A96 | Meissner K, Ziep D (2011). Organ-specifity of placebo effects on blood pressure. Autonomic Neuroscience, 164:62–66. |
| A97 | Merklinger S, Messemer C, Niederle S (1995). Ekzembehandlung mit cardiospermum halicacabum: Cardiospermum-Salbe und Salbengrundlage im Halbseitenvergleich - eine kontrolllerte Studie [Treatment of eczema with Cardiospermum halicacabum: Cardiospermum ointment and the ointment vehicle - a controlled study]. Zeitschrift für Phytotherapie; 16:263–266. |
| A98 | Mokkapatti R (1992). An experimental double-blind study to evaluate the use of Euphrasia in preventing conjunctivitis. British Homoeopathic Journal; 81:22–24. |
| A99 | Mousavi F, Sherafati S, Mojaver YN (2009). Ignatia in the treatment of oral lichen planus. Homeopathy; 98:40–44. |
| A100 | Oberbaum M, Yaniv I, Ben-Gal Y, Stein J, Ben-Zvi N, Freedman LS, Branski D (2001). A randomized, controlled clinical trial of the homeopathic medication Traumeel S in the treatment of chemotherapy-induced stomatitis in children undergoing stem cell transplantation. Cancer; 92:684–690. |
| A101 | Oberbaum M, Galoyan N, Lerner-Geva L, Singer SR, Grisaru S, Shashar D, Samueloff A (2005). The effect of the homeopathic remedies Arnica and Bellis perennis on mild postpartum bleeding – a randomized, double-blind, placebo-controlled study – preliminary results. Complementary Therapies in Medicine; 13:87–90. |
| A102 | Pach D, Brinkhaus B, Roll S, Wegscheider K, Icke K, Willich SN, Witt CM (2011). Efficacy of injections with Disci/Rhus toxicodendron compositum for chronic low back pain - A randomized placebo-controlled trial. PLoS One; 6: e26166. Epub 2011 Nov 8. |
| A103 | Padilha RQ, Riera R, Atallah ÁN (2011). Homeopathic Plumbum metallicum for lead poisoning: a randomized clinical trial. Homeopathy;100:116–121. |
| A104 | Papp R, Schuback G, Beck E, Burkard G, Bengel J, Lehrl S, Belon P (1998). Oscillococcinum® in patients with influenza-like syndromes: a placebo-controlled double-blind evaluation. British Homoeopathic Journal; 87:69–76. |
| A105 | Paris A, Gonnet N, Chaussard C, Belon P, Rocourt F, Saragaglia D, Cracowski JL (2008). Effect of homeopathy on analgesic intake following knee ligament reconstruction: a phase III monocentre randomized placebo controlled study. British Journal of Clinical Pharmacology; 65:180–187. |
| A106 | Paris A, Schmidlin S, Mouret S, Hodaj E, Marijnen P, Boujedaini N, Polosan M, Cracowski JL (2011). Effect of Gelsemium 5CH and 15CH on anticipatory anxiety: A phase III, single-centre, randomized, placebo-controlled study. Fundamental and Clinical Pharmacology; 25:42. |
| A107 | Plezbert JA, Burke JR (2005). Effects of the homeopathic remedy Arnica on attenuating symptoms of exercise-induced muscle soreness. Journal of Chiropractic Medicine; 4:152–161. |
| A108 | Rahlfs VW, Mössinger P (1976). Zur Behandlung des Colon irritable: Ein multizentrischer plazebo-kontrollierter Doppelblindversuch in der Allgemeinen Praxis [Treatment of irritable colon: A multicenter placebo-controlled double-blind study in general practice]. Arzneimittel Forschung; 26:2230–2234. |
| A109 | Rahlfs VW, Mössinger P (1978). Asa foetida bei Colon irritabile – Doppelblindversuch [Asa foetida in the treatment of the irritable colon – a double-blind trial]. Deutsche medizinische Wochenschrift; 104:140–143. |
| A110 | Ramelet AA, Buchheim G, Lorenz P, Imfeld M (2000). Homeopathic Arnica in postoperative haematomas: a double-blind study. Dermatology; 201: 347–348. |
| A111 | Reilly DT, Taylor MA, McSharry C, Aitchison T (1986). Is homeopathy a placebo response? Controlled trial of homeopathic potency, with pollen in hayfever as model. Lancet; ii:881–885. |
| A112 | Reilly D, Taylor MA, Beattie NGM, Campbell JH, McSharry C, Aitchison TC, Carter R, Stevenson RD (1994). Is evidence for homeopathy reproducible? Lancet; 344:1601–1606. |
| A113 | Robertson A, Suryanarayanan R, Banerjee A (2007). Homeopathic Arnica montana for post-tonsillectomy analgesia: a randomised placebo control trial. Homeopathy; 96:17–21. |
| A114 | Saruggia M, Corghi E (1992). Effects of homoeopathic dilutions of china rubra on intradialytic symptomatology in patients treated with chronic haemodialysis. British Homoeopathic Journal; 81:86–88. |
| A115 | Schirmer K-P, Fritz M, Jäckel WH (2000). Wirksamkeit von Formica rufa und Eigenblut-Injektionen bei Patienten mit ankylosierender Spondylitis: eine doppelblinde, randomisierte Studie [Efficacy of Formica rufa and reinjection of patient's own blood on patients with ankylosing spondylitis: a double-blind, randomized study]. Zeitschrift für Rheumatologie; 59:321–329. |
| A116 | Schmidt JM, Ostermayr B (2002). Does a homeopathic ultramolecular dilution of Thyroidinum 30cH affect the rate of body weight reduction in fasting patients? A randomized placebo-controlled double-blind clinical trial. Homeopathy; 91:197–206. |
| A117 | Seeley BM, Denton AB, Ahn MS, Maas CS (2006). Effect of homeopathic Arnica montana on bruising in face-lifts: results of a randomized, double-blind, placebo-controlled clinical trial. Archives of Facial Plastic Surgery; 8:54–59. |
| A118 | Shipley M, Berry H, Broster G, Jenkins M, Clover A, Williams I (1983). Controlled trial of homoeopathic treatment of osteoarthritis. Lancet; i:97–98. |
| A119 | Simpson JJ, Donaldson I, Davies WE (1998). Use of homeopathy in the treatment of tinnitus. British Journal of Audiology; 32:227–233. |
| A120 | Singer SR, Amit-Kohn M, Weiss S, Rosenblum J, Maoz G, Samuels N, Lukasiewicz E, Freedman L, Paltiel O, Itzchaki M, Niska M, Oberbaum M (2010). Traumeel S for pain relief following hallux valgus surgery: a randomized controlled trial. BMC Clinical Pharmacology; 10: 9. |
| A121 | Smith SA, Baker AE, Williams JH (2002). Effective treatment of seborrhoeic dermatitis using a low dose, oral homeopathic medication consisting of potassium bromide, sodium bromide, nickel sulfate, and sodium chloride in a double-blind, placebo-controlled study. Alternative Medicine Review; 7:59–67. |
| A122 | Stevinson C, Devaraj VS, Fountain-Barber A, Hawkins S, Ernst E (2003). Homeopathic arnica for prevention of pain and bruising: randomized placebo-controlled trial in hand surgery. Journal of the Royal Society of Medicine; 96:60–65. |
| A123 | Taylor MA, Reilly D, Llewellyn-Jones RH, McSharry C, Aitchison TC (2000). Randomised controlled trial of homoeopathy versus placebo in perennial allergic rhinitis with overview of four trial series. British Medical Journal; 321:471–476. |
| A124 | Tuten C, McLung J (1999). Reducing muscle soreness with Arnica montana. Alternative and Complementary Therapies; 5:369–372. |
| A125 | Tveiten D, Bruseth S, Borchgrevink CF, Løhne K (1991). Effekt av Arnica D 30 ved hard fysisk anstrengelse. En doppeltblind randomisert undersøkelse under Oslo maraton 1990 [Effect of Arnica D30 on hard physical exertion. A double-blind randomized trial during the 1990 Oslo Marathon]. Tidsskrift for den Norske Laegeforening, 111:3630–3631. |
| A126 | Tveiten D, Bruseth S, Borchgrevink CF, Norseth J (1998). Effects of the homoeopathic remedy Arnica D30 on marathon runners: a randomized, double-blind study during the 1995 Oslo Marathon. Complementary Therapies in Medicine, 6:71–74. |
| A127 | Vickers AJ, Fisher P, Smith C, Wyllie SE, Lewith GT (1997). Homoeopathy for delayed onset muscle soreness: a randomized double blind placebo controlled trial. British Journal of Sports Medicine, 31:304–307. |
| A128 | Vickers AJ, Fisher P, Smith C, Wyllie SE, Rees R (1998). Homeopathic Arnica 30X is ineffective for muscle soreness after long-distance running: a randomized, double-blind, placebo-controlled trial. Clinical Journal of Pain, 14:227–231. |
| A129 | von Hagens C, Schiller P, Godbillon B, Osburg J, Klose C, Limprecht R, Strowitzki T (2012). Treating menopausal symptoms with a complex remedy or placebo: a randomized controlled trial. Climacteric; 15:358–367 [Epub ahead of print, 2011]. |
| A130 | Weiser M, Clasen B (1994). Randomisierte plazebokontrolierte Doppelblindstudie zur Untersuchung der klinische Wirksamkeit der homöopathischen Euphorbium compositum-Nasentropfen S bei chronischer Sinusitis [Randomized, placebo-controlled, double-blind study of the clinical efficacy of the homeopathic Euphorbium compositum-S nasal spray in cases of chronic sinusitis]. Forschende Komplementärmedizin; 1:251–259. |
| A131 | Wiesenauer M, Gaus W (1985). Double-blind trial comparing the effectiveness of the homoeopathic preparation Galphimia potentization D6, Galphimia dilution 10⁻6 and placebo on pollinosis. Arzneimittel Forschung; 35:1745–1747. |
| A132 | Wiesenauer M, Gaus W, Bohnacker U, Häussler S (1989). Wirksamkeitsprüfung von homöopathische Kombinationspräparaten bei Sinusitis. Ergebnisse einer randomisierten Doppelblindstudie unter Praxisbedingungen [Efficiency of homeopathic preparation combinations in sinusitis. Results of a randomized double blind study with general practitioners]. Arzneimittel Forschung; 39:620-625. |
| A133 | Wiesenauer M, Gaus W, Häussler S (1990). Behandlung der Pollinoisis mit Galphimia glauca. Eine Doppelblindstudie unter Praxisbedingungen [Treatment of pollinosis with the homeopathic preparation Galphimia glauca. A double-blind trial in clinical practice]. Allergologie; 13:359–363. |
| A134 | Wiesenauer M, Gaus W (1991). Wirksamkeitsnachweis eines Homöopathikums bei chronischer Polyarthritis. Eine randomisierte Doppelblindstudie bei nieder-gelassenen Ärzten [A randomized double-blind trial on the efficacy of a homeopathic drug for rheumatoid arthritis]. Aktuelle Rheumatologie; 16:1–21. |
| A135 | Wiesenauer M, Lüdtke R (1995). The treatment of pollinosis with Galphimia glauca D4 - a randomized placebo-controlled double-blind clinical trial. Phytomedicine; 2:3–6. |
| A136 | Wolf M, Tamaschke C, Mayer W, Heger M (2003). Wirksamkeit von Arnica bei Varizenoperation: Ergebnisse einer randomisierten, doppelblinden, Placebo-kontrollierten Pilot-Studie [Efficacy of Arnica in varicose vein surgery: results of a randomized, double-blind, placebo-controlled pilot study]. Forschende Komplementärmedizin und Klassische Naturheilkunde; 10:242–247. |
| A137 | Zabolotnyi DI, Kneis KC, Richardson A, Rettenberger R, Heger M, Kaszkin-Bettag M, Heger PW (2007). Efficacy of a complex homeopathic medication (Sinfrontal) in patients with acute maxillary sinusitis: a prospective, randomized, double-blind, placebo-controlled, multicenter clinical trial. Explore (NY); 3:98–109. |
| A269 | Bell IR, Howerter A, Jackson N, Brooks AJ, Schwartz GE (2012). Multiweek resting EEG cordance change patterns from repeated olfactory activation with two constitutionally salient homeopathic remedies in healthy young adults. Journal of Alternative and Complementary Medicine; 18:445–453. |
| A270 | Bell IR, Howerter A, Jackson N, Aickin M, Bootzin RR, Brooks AJ (2012). Nonlinear dynamical systems effects of homeopathic remedies on multiscale entropy and correlation dimension of slow wave sleep EEG in young adults with histories of coffee-induced insomnia. Homeopathy; 101:182–192. |
| A271 | Bell IR, Brooks AJ, Howerter A, Jackson N, Schwartz GE (2013). Acute electroencephalographic effects from repeated olfactory administration of homeopathic remedies in individuals with self-reported chemical sensitivity. Alternative Therapies in Health and Medicine; 19:46–57. |
| A272 | Colau JC, Vincent S, Marijnen P, Allaert FA (2012). Efficacy of a non-hormonal treatment, BRN-01, on menopausal hot flashes: a multicenter, randomized, double-blind, placebo-controlled trial. Drugs R D; 12:107–119. |
| A273 | Dean ME, Karsandas R, Bland JM, Gooch D, MacPherson H (2012). Homeopathy for mental fatigue: lessons from a randomized, triple blind, placebo-controlled cross-over clinical trial. BMC Complementary and Alternative Medicine; 12:167. |
| A274 | Harrison CC, Solomon EM, Pellow J (2013). The effect of a homeopathic complex on psychophysiological onset insomnia in males: a randomized pilot study. Alternative Therapies in Health and Medicine; 19:38–43. |
| A275 | Naidoo P, Pellow J (2013). A randomized placebo-controlled pilot study of Cat saliva 9cH and Histaminum 9cH in cat allergic adults. Homeopathy; 102:123–129. |
| A276 | Pérol D, Provençal J, Hardy-Bessard AC, Coeffic D, Jacquin JP, Agostini C, Bachelot T, Guastalla JP, Pivot X, Martin JP, Bajard A, Ray-Coquard I (2012). Can treatment with Cocculine improve the control of chemotherapy-induced emesis in early breast cancer patients? A randomized, multi-centered, double-blind, placebo-controlled Phase III trial. BMC Cancer; 12:603. |
| A277 | Razlog R, Pellow J, White SJ (2012). A pilot study on the efficacy of Valeriana officinalis mother tincture and Valeriana officinalis 3x in the treatment of attention deficit hyperactivity disorder. Health SA Gesondheid; 17; #603. |
| A278 | Sencer SF, Zhou T, Freedman LS, Ives JA, Chen Z, Wall D, Nieder ML, Grupp SA, Yu LC, Sahdev I, Jonas WB, Wallace JD, Oberbaum M (2012). Traumeel S in preventing and treating mucositis in young patients undergoing SCT: a report of the Children's Oncology Group. Bone Marrow Transplant; 47:1409–1414. |
| A279 | Zanasi A, Mazzolini M, Tursi F, Morselli-Labate AM, Paccapelo A, Lecchi M (2014). Homeopathic medicine for acute cough in upper respiratory tract infections and acute bronchitis: A randomized, double-blind, placebo-controlled trial. Pulmonary Pharmacology and Therapeutics; 27:102–108 [Epub ahead of print, 2013]. |
| A293 | Malapane E, Solomon EM, Pellow J (2014). Efficacy of a homeopathic complex on acute viral tonsillitis. Journal of Alternative and Complementary Medicine; 20:868–873. |
| A294 | Patil AS, Jadhav AB, Arya MP (2014). Effect of biochemic preparation of ferrum phos 3x on blood neutrophils. Der Pharmacia Lettre; 6:169–171. |
| A295 | Nair KRJ, Gopinadhan S, Kurup TNS, Kumar BSJR, Aggarwal A, Varanasi R, Nayak D, Padmanabhan M, Oberai P, Singh H, Singh VP, Nayak C (2014). Homoeopathic genus epidemicus 'Bryonia alba' as a prophylactic during an outbreak of Chikungunya in India: A cluster-randomised, double-blind, placebo-controlled trial. Indian Journal of Research in Homeopathy; 8:160–165. |
