# Supplementary figures and images for: Randomised, double-blind, placebo-controlled trials of non-individualised homeopathic treatment: systematic review and meta-analysis
Source: Syst Rev. 2017 Mar 24;6:63. doi: 10.1186/s13643-017-0445-3 (PMC5366148; doi:10.1186/s13643-017-0445-3)

**Additional file 3**


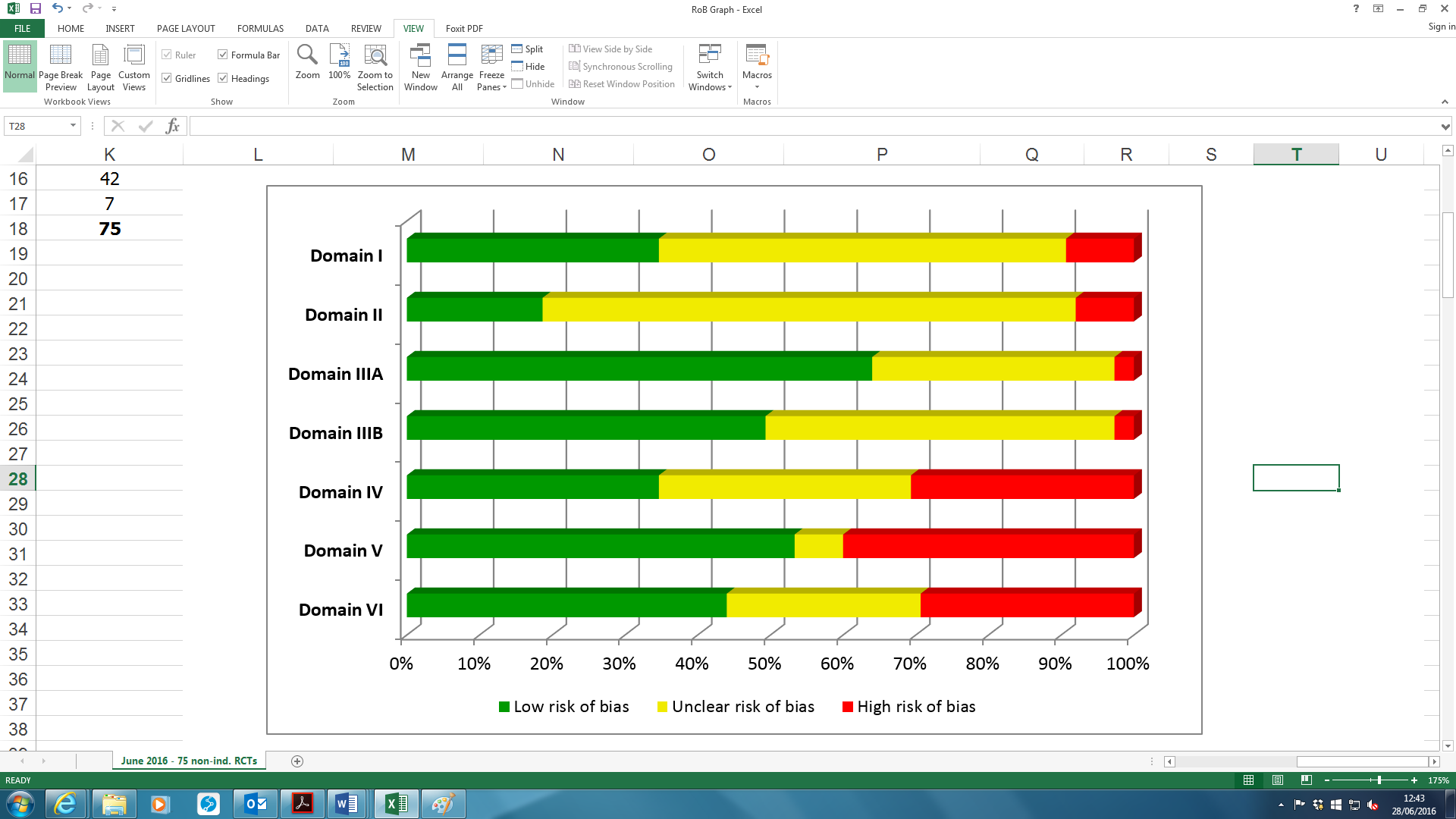

Supplement: Supplementary file 3 — Risk-of-bias bar-graph for 75 RCTs of non-individualised homeopathy. (DOCX 170 kb) [file 13643_2017_445_MOESM3_ESM.docx]

RE Model

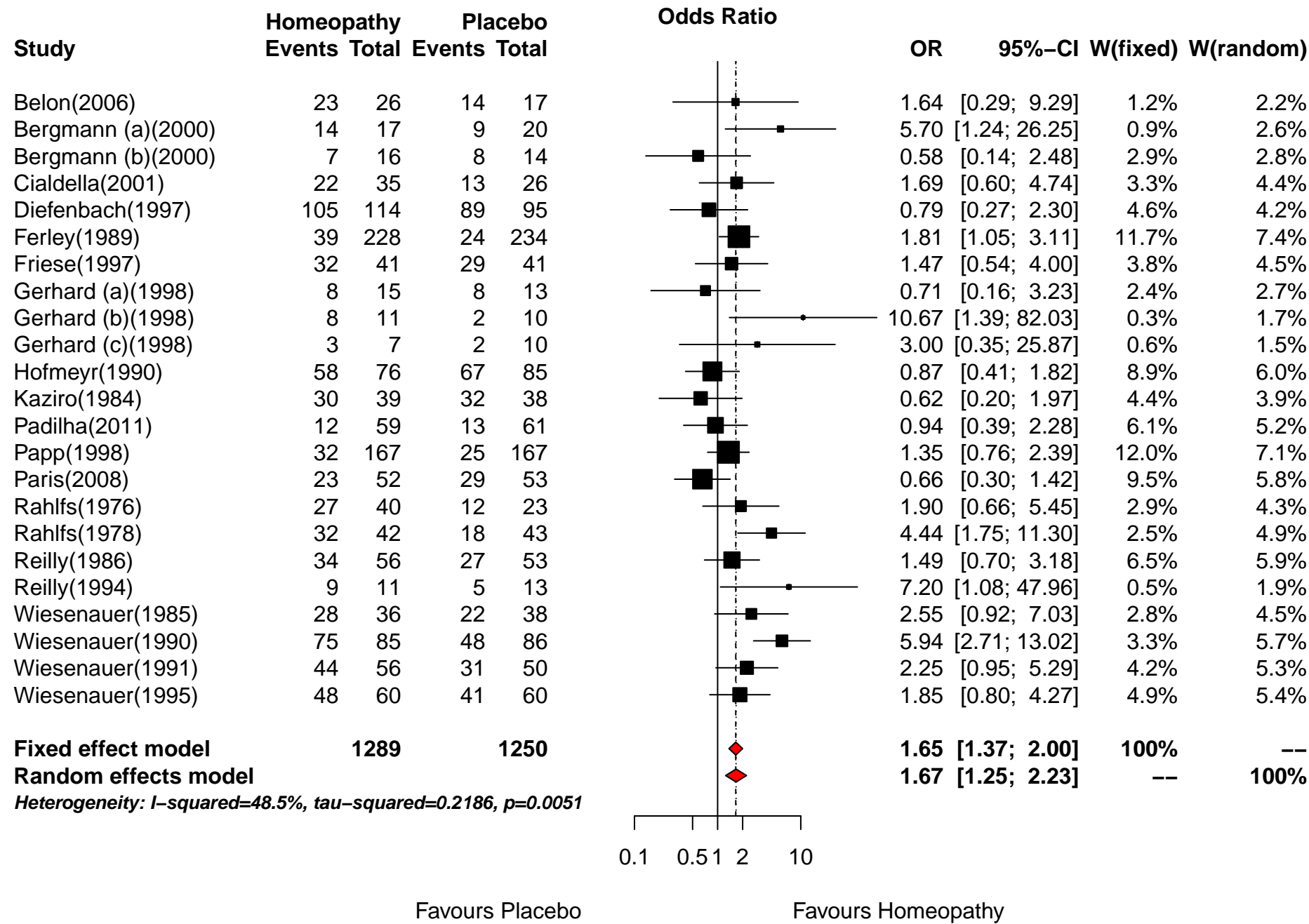

Supplement: Supplementary file 4 — Forest plots, showing (a) standardised mean difference (SMD) and (b) odds ratio (OR), with 95% confidence interval (CI) for original data (continuous or dichotomous) extracted per trial of non-individualised homeopathy. Pooled effects estimate shown for fixed-effect and random-effects model. W, weighting. To ensure consistent direction of measurement with disease severity, sign inversion was applied to the mean value of five trials in (a). [Reflecting the fact that OR > 1 favours homeopathy, the direction of change toward homeopathy in plot (b) is to the right, thus differing from all other plots]. (ZIP 15 kb) [file 13643_2017_445_MOESM4_ESM.zip › SR2 - Additional file 4b - dcs_forest_plot_v2R3.pdf]
