# Supplementary material for: Randomised, double-blind, placebo-controlled trials of non-individualised homeopathic treatment: systematic review and meta-analysis
Source: Syst Rev. 2017 Mar 24;6:63. doi: 10.1186/s13643-017-0445-3 (PMC5366148; doi:10.1186/s13643-017-0445-3)

RE Model

Study	Homeopathy Total	Homeopathy Mean	Homeopathy SD	Placebo Total	Placebo Mean	Placebo SD	SMD	95%−CI	W(fixed)	W(random)
Baker(2003)	21	64.10	13.50	23	58.70	11.50	0.42	[−0.17; 1.02]	1.8%	3.0%
Balzarini(2000)	29	2.35	1.34	32	3.25	2.08	−0.50	[−1.01; 0.01]	2.4%	3.4%
Beer(1999)	20	13.00	11.80	20	13.40	10.10	−0.04	[−0.66; 0.58]	1.7%	2.9%
Bignamini(1987)	17	160.00	20.20	15	161.00	25.90	−0.04	[−0.74; 0.65]	1.3%	2.7%
Clark(2000)	7	13.75	23.37	7	45.75	23.42	−1.28	[−2.47; −0.09]	0.5%	1.4%
Colau(2012)	50	82.30	49.40	51	113.00	88.20	−0.43	[−0.82; −0.03]	4.1%	3.9%
Cornu(2010)	46	640.00	297.00	46	796.00	717.00	−0.28	[−0.69; 0.13]	3.8%	3.8%
Ernst(1990)	62	−34.40	23.60	60	−26.10	17.00	−0.40	[−0.76; −0.04]	5.0%	4.1%
Frass(2005)	25	1.52	0.59	25	2.44	0.65	−1.46	[−2.09; −0.83]	1.6%	2.9%
Freitas(1995)	38	7.55	7.83	31	9.02	8.63	−0.18	[−0.65; 0.30]	2.8%	3.6%
GRECHO(1989)	150	98.80	42.00	150	94.40	40.70	0.11	[−0.12; 0.33]	12.5%	4.6%
Jacobs(2007)	28	3.71	1.90	30	3.58	1.70	0.07	[−0.44; 0.59]	2.4%	3.4%
Khuda−Bukhsh(2005)	33	−71.00	57.40	22	−58.00	28.10	−0.27	[−0.81; 0.27]	2.2%	3.3%
Khuda−Bukhsh(2011)	9	−54.08	10.64	5	−50.72	11.50	−0.29	[−1.39; 0.81]	0.5%	1.6%
Kim(2005)	18	1.85	1.15	16	2.25	0.93	−0.37	[−1.05; 0.31]	1.4%	2.7%
Kotlus(2010)	28	0.87	0.84	29	1.09	1.18	−0.21	[−0.73; 0.31]	2.4%	3.4%
Leaman(1989)	17	5978.00	4172.00	17	6946.00	3215.00	−0.25	[−0.93; 0.42]	1.4%	2.7%
Lewith(2002)	101	2.44	1.51	101	2.64	1.61	−0.13	[−0.40; 0.15]	8.4%	4.4%
Lipman(1999)	44	−1.58	1.20	46	−0.73	0.94	−0.78	[−1.21; −0.35]	3.5%	3.8%
Malapane(2014)	15	1.60	2.03	15	5.20	3.00	−1.37	[−2.17; −0.56]	1.0%	2.3%
Naidoo(2013)	15	2.27	1.87	15	5.67	2.64	−1.45	[−2.26; −0.63]	1.0%	2.3%
Oberbaum(2001)	15	10.03	11.55	15	24.27	14.86	−1.04	[−1.81; −0.27]	1.1%	2.4%
Oberbaum(2005)	28	−12.40	2.43	12	−11.60	2.12	−0.33	[−1.02; 0.35]	1.4%	2.7%
Robertson(2007)	53	5.50	6.80	58	9.00	11.40	−0.37	[−0.74; 0.01]	4.5%	4.0%
Singer(2010)	39	55.40	25.50	40	57.40	25.70	−0.08	[−0.52; 0.36]	3.3%	3.7%
Taylor (2000)	23	34.50	12.90	27	34.10	18.70	0.02	[−0.53; 0.58]	2.1%	3.2%
Tveiten(1991)	20	0.80	1.37	16	1.40	1.43	−0.42	[−1.09; 0.25]	1.4%	2.8%
Tveiten(1998)	24	12.50	15.00	22	12.20	12.20	0.02	[−0.56; 0.60]	1.9%	3.1%
Vickers(1998)	200	45.20	23.90	200	41.00	23.60	0.18	[−0.02; 0.37]	16.6%	4.7%
Wolf(2003)	30	38.60	59.50	29	63.40	127.70	−0.25	[−0.76; 0.27]	2.4%	3.4%
Zabolotnyi(2007)	56	8.70	2.62	57	11.90	2.34	−1.28	[−1.69; −0.87]	3.9%	3.9%
**Fixed effect model**	**1261**			**1232**			**−0.23**	**[−0.31; −0.15]**	**100%**	**−−**
**Random effects model**							**−0.36**	**[−0.52; −0.19]**	**−−**	**100%**

Heterogeneity: I−squared=72.3%, tau−squared=0.1397, p<0.0001

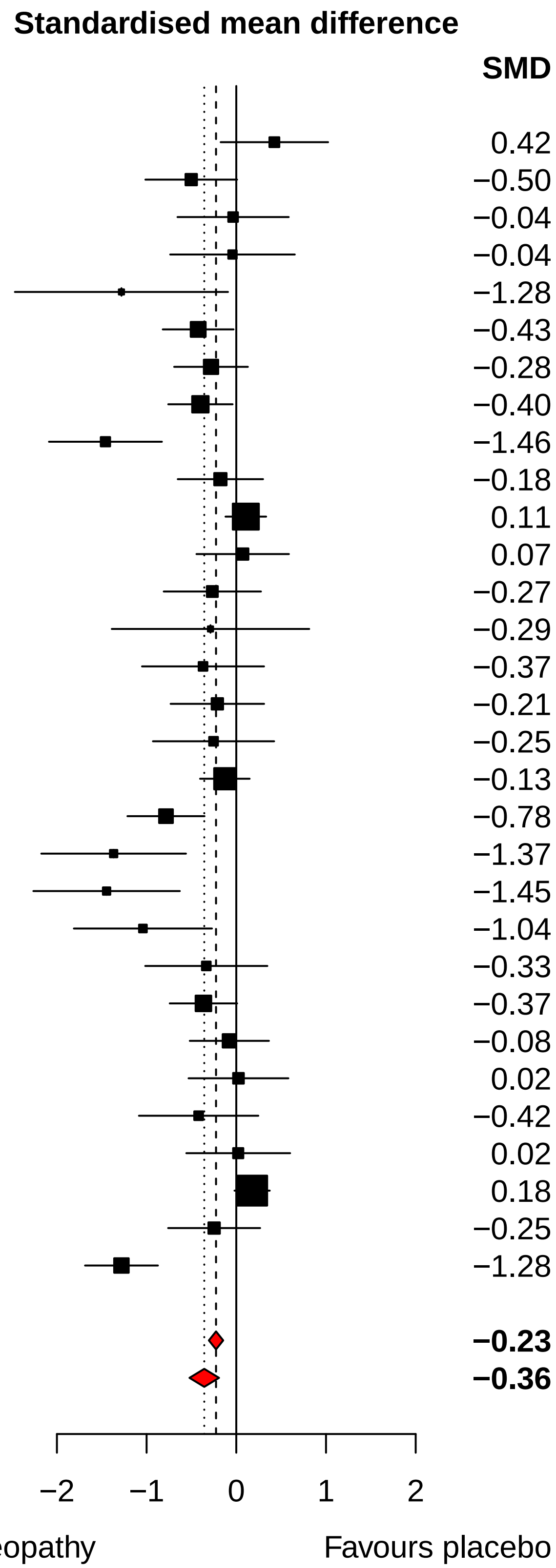

Supplement: Supplementary file 4 — Forest plots, showing (a) standardised mean difference (SMD) and (b) odds ratio (OR), with 95% confidence interval (CI) for original data (continuous or dichotomous) extracted per trial of non-individualised homeopathy. Pooled effects estimate shown for fixed-effect and random-effects model. W, weighting. To ensure consistent direction of measurement with disease severity, sign inversion was applied to the mean value of five trials in (a). [Reflecting the fact that OR > 1 favours homeopathy, the direction of change toward homeopathy in plot (b) is to the right, thus differing from all other plots]. (ZIP 15 kb) [file 13643_2017_445_MOESM4_ESM.zip › SR2 - Additional file 4a - cts_forest_plot2R3.pdf]
